# Comparing FIB-4, VCTE, pSWE, 2D-SWE, and MRE Thresholds and Diagnostic Accuracies for Detecting Hepatic Fibrosis in Patients with MASLD: A Systematic Review and Meta-Analysis

**DOI:** 10.3390/diagnostics15131598

**Published:** 2025-06-24

**Authors:** Mitchell Patrick Wilson, Ranjit Singh, Shyam Mehta, Mohammad Hassan Murad, Christopher Fung, Gavin Low

**Affiliations:** 1 Department of Radiology and Diagnostic Imaging, University of Alberta, 8440-112 Street NW, Edmonton, AB T6G 2B7, Canadasmehta1@ualberta.ca (S.M.);; 2 Evidence-Based Practice Center, Mayo Clinic, Rochester, MN 55905, USA

**Keywords:** metabolic dysfunction-associated steatotic liver disease, MASLD, transient elastography, fibroScan, shear wave elastography, SWE, magnetic resonance elastography, MRE, accuracy, systematic review

## Abstract

**Objectives**: To compare thresholds and accuracies of FIB-4, vibration-controlled transient elastography (VCTE), point shear wave elastography (pSWE), 2D shear wave elastography (2D-SWE), and MR elastography (MRE) for detecting hepatic fibrosis in patients with MASLD. **Materials and Methods**: Systematic searching of MEDLINE, EMBASE, Cochrane Library, Scopus, and the gray literature from inception to March 2024 was performed. Studies evaluating accuracies of FIB-4, VCTE, 2D-SWE, pSWE, and/or MRE for detecting significant (≥F2) and/or advanced (≥F3) hepatic fibrosis in MASLD patients compared to histology were identified. Full-text review and data extraction were performed independently by two reviewers. Multivariate meta-analysis and subgroup analyses were performed using index test and fibrosis grading. Risk of bias was assessed using QUADAS-2. **Results**: 207 studies with over 80,000 patient investigations were included. FIB-4 1.3 threshold sensitivity was 71% (95% CI 66–75%) for detecting advanced hepatic fibrosis, which improved to 88% (85–91%) using a <0.75 threshold. FIB-4 specificity using a 2.67 threshold was 96% (94–97%). Sensitivities of 88–91% were achieved using thresholds of 3.2 kPa for pSWE, 4.92 kPa for 2D-SWE, 7.18 kPa for VCTE, and 2.32 kPa for MRE. No significant differences were identified for sensitivities in subgroup analysis with thresholds between 7 and 9 kPa. Most imaging-based studies were high risk of bias for the index test. **Conclusions**: A FIB-4 threshold of <0.75 and modality-dependent thresholds (VCTE < 7 kPa; pSWE <3 kPa; 2D-SWE <5 kPa; and MRE <2.5 kPa) would achieve sensitivities of around 90% when defining low-risk MASLD in population screening. A modified two-tier algorithm aligning with existing Society of Radiologists in Ultrasound guidelines would improve risk stratification accuracies compared to existing guidelines by European and American liver societies.

## 1. Introduction

Metabolic dysfunction-associated steatotic liver disease (MASLD), formerly non-alcoholic fatty liver disease (NAFLD), has become the most common cause of chronic liver disease (CLD) in the Western world with a rising incidence [1]. MASLD consists of a spectrum of steatotic liver diseases ranging from isolated liver steatosis (metabolic dysfunction-associated steatotic liver [MASL]), hepatocellular ballooning and lobular inflammation (metabolic dysfunction-associated steatohepatitis [MASH]), and hepatic fibrosis ultimately to cirrhosis. Hepatic fibrosis and cirrhosis are associated with liver failure, portal hypertension and other complications including hepatocellular carcinoma, ultimately resulting in approximately 2 million deaths annually worldwide [2]. Early detection and risk stratification of patients on the MASLD spectrum can help identify patients who may benefit from lifestyle and medical intervention to prevent and even reverse early stages of CLD. The traditional gold standard, liver biopsy, is subject to several limitations restricting widespread use at the population level. As such, several alternative non-invasive approaches to detect significant and advanced liver fibrosis have been developed, including various combinations of clinical and laboratory investigations (the most popular calculation now used is termed Fibrosis-4 [FIB-4]), as well as several imaging-based tools such as vibration-controlled transient elastography (VCTE), point shear wave elastography (pSWE), 2D shear wave elastography (2D-SWE), and MR elastography (MRE). These elastography technologies utilize different physical properties including variations of vibration-controlled technologies (TE and MRE) and acoustic radiation force impulses (pSWE and 2D-SWE).

Several guidelines have recently been published offering recommendations for population-level risk stratification using non-invasive techniques [1,2,3,4]. These guidelines have included a range of recommendations from a single vendor agnostic image-based multi-threshold risk stratification tool independent of underlying pathophysiology [4] to variations of a two-step blood-based followed by imaging-based vendor and modality agnostic approach, to risk stratification thresholds [1,3] and disease severity grading [2]. In guidelines using multi-modality image-based risk stratification tools, only one differentiated thresholds for MRE from other liver stiffness measurement (LSM) techniques [2] but did not differentiate between VCTE, pSWE, nor 2D-SWE. Given that each technique differs fundamentally in underlying technology, a nuanced evaluation of accuracy is needed, comparing the accuracy and threshold grading of these modalities to inform current and future revisions of these population-level risk stratification guidelines. This meta-analysis aims to evaluate the individual diagnostic accuracies of FIB-4, VCTE, pSWE, 2D-SWE, and MRE for detecting significant (METAVIR ≥ F2) and advanced (METAVIR ≥ F3) hepatic fibrosis in patients with MASLD at differing thresholds. Subgroup analyses are performed to determine the accuracies of frequently recommended modality-specific thresholds.

## 2. Methods

This systematic review and meta-analysis were reported in accordance with the Preferred Reporting Items for Systematic Reviews and Meta-Analysis–Diagnostic Test Accuracy (PRISMA-DTA) guidelines (Supplementary Table S1) [5]. Prior to initiation, a study proposal was submitted to the PROSPERO database (CRD42024528716). Institutional ethics approval and patient consent were not required as this review included pooled analysis of previously published studies.

### 2.1. Literature Search

A systematic search of multiple databases was performed to identify studies evaluating the diagnostic accuracy of one or more of (1) FIB-4, (2) VCTE, (3) pSWE, (4) 2D-SWE, or (5) MRE for detecting significant and/or advanced hepatic fibrosis in patients with MASLD. Individualized search protocols for MEDLINE, EMBASE, Cochrane Library (systematic reviews and registry of controlled trials), and Scopus from the date of inception up to March 25, 2024 were developed by a reviewer with 10 years of imaging experience and expertise in the performance of diagnostic test accuracy systematic reviews (M.P.W.). Several combinations of title/abstract/keywords and medical subject headings pertaining to information related to patient population (NAFLD/MASLD), index test(s) (FIB-4, VCTE, pSWE, 2D-SWE, and MRE) and diagnostic accuracy were customized by database. Individualized database search criteria are shown in Appendix A. No language restriction was applied. The electronic search was conducted according to best practices [6]. Studies from individual databases were then collated, and duplicates were removed. A title and abstract review was performed by a separate reviewer with 10 years of imaging experience and experience in diagnostic test accuracy studies (R.S.). A full-text review was then conducted separately in a blinded fashion by this reviewer and a medical student with prior experience in scoping reviews (R.S., S.M.), with a gray literature search performed in tandem by S.M. evaluating the most recent three years of meetings by the Radiological Society of North America (RSNA), the American Roentgen Ray Society (ARRS), and the American College of Gastroenterology (ACG). References for relevant articles were manually evaluated by reviewers, and forward searching of relevant included articles was also performed on Google Scholar.

### 2.2. Selection Criteria

Studies were identified and ultimately selected for inclusion when evaluating one or more index test diagnostic accuracies using the following criteria: (1) MASLD patient population; (2) one or more of FIB-4, VCTE, pSWE, 2D-SWE, and/or MRE used as the index test; (3) histopathology used as the reference standard; (4) studies evaluating a minimum of 5 patients; and (5) sufficient information to construct a 2 × 2 contingency table (true positive [TP], false positive [FP], false negative [FN], and true negative [TN]). Histopathology reference standards were referenced to METAVIR 0–1 versus 2–4 (“significant hepatic fibrosis”) and/or METAVIR 0–2 versus 3–4 (“advanced hepatic fibrosis”). When studies used alternative histopathology staging systems for hepatic fibrosis (such as Ishak score or Batts–Ludwig system), studies were correlated to METAVIR grading using previously proposed standardized correspondence between systems [7]. Studies were excluded from review if (1) MASLD was not the selected patient population and/or the performance of index tests in only MASLD patients could not be extracted from pooled data with other patient populations; (2) an index test other than the above was used; (3) patients without a histopathological reference standard were included; and (4) they were non-original articles, including review articles, guidelines, consensus statements, letters, and editorials.

### 2.3. Data Extraction

Two reviewers (R.S., S.M.) independently extracted data. Study, patient, and index test characteristics were recorded, including author, year, country of institution, study design (prospective or retrospective), number of centers involved in patient recruitment (single- or multicenter), reference standard, total number of patients, mean age and range, percentage of male sex, total number of cases of METAVIR ≥ 2 and/or ≥ 3, index test(s) used, thresholds by index test and fibrosis severity, reader agreement, and pertinent notes by study. For studies evaluating one or more of pSWE, 2D-SWE, and/or VCTE, details related to the index test including vendor used, probe frequency (when applicable), number of acquisitions of elastography, and technician (person performing the examination) experience were extracted. For studies evaluating MRE as an index test, details including vendor used, reader experience, MRI field strength (1.5 T or 3 T), pulse sequence used (gradient echo [GRE] versus spin echo echoplanar imaging [SE-EPI]), passive driver amplitude, and segmentation technique used (manual/freehand or automated) were extracted. Contingency tables were developed for each individual index test and fibrosis severity, and corresponding thresholds were recorded. Thresholds were reported as Young’s modulus (kPa) for imaging-based modalities. For studies reporting elasticity with shear wave velocities (m/s), a conversion calculation of E = 3*p*c^2^ was used, where E = Young’s modulus, *p* = density [1000 kg/m^3^], and c = shear wave velocity [8]. When true positive, false positive, false negative, and true negative cases were not reported directly, a contingency table was constructed using the total number of patients included in that index test, the total number of positive cases (based on fibrosis severity), and sensitivity and specificity. If contingency tables were provided by multiple readers within a single index test and fibrosis severity, results were averaged as a single result based on the a priori design [9]. After independent extraction, the data were combined into a single file where discrepancies were resolved by re-review and consensus between the two data extractors and a third author (R.S., S.M., and M.P.W.).

### 2.4. Risk of Bias Assessment

Risk of bias and applicability concerns were evaluated on a per study basis using the Quality Assessment of Diagnostic Accuracy Studies-2 (QUADAS-2) tool [10]. The patient selection, reference standard, and flow and timing were assessed using standardized signaling questions outlined in QUADAS-2. Any single signaling question marked as high risk would result in a high-risk grading for a given domain.

### 2.5. Data Analysis

A bivariate mixed-effects regression model was initially planned in an a priori protocol with stratification by index test, fibrosis severity, and threshold used. However, after extraction of the data and recognition of inconsistent threshold performance variably reported across a continuous spectrum by study and index test, a multilevel random effects model was instead performed with stratification by index test and fibrosis severity (METAVIR 0–1 versus 2–4 and/or 0–2 versus 3–4) for the primary analysis. This model links the range of thresholds and retrospective pairs of sensitivity and specificity to identify thresholds at which the test is likely to perform optimally. The model assumes a logistic distribution and estimates the distribution parameters in patients without significant and/or advanced hepatic fibrosis, applying a linear mixed-effects model to the transformed data. The model accounts for between-study variability and dependence of sensitivity and specificity. Three separate sensitivity weightings were applied (sensitivity to specificity weights of 0.3:0.7, 0.5:0.5, and 0.7:0.2). These weightings were selected to recognize likely “rule in”, “balanced”, and “rule out” thresholds, respectively. Continuity correction using a value of one was applied to any cells in a 2 × 2 contingency table where a zero value was encountered. Summary sensitivities and specificities with a 95% confidence interval (CI) were estimated for each threshold. A separate multilevel random effects model was applied for FIB-4 studies evaluating thresholds of 1.3 and 2.67 for detecting both significant and advanced hepatic fibrosis based on current guideline recommendations. Finally, a vendor-specific multilevel random effects model was applied for pSWE, 2D-SWE, and VCTE studies in the same fashion.

Statistical measures of study variability and publication bias were not assessed as these are proven to be unreliable and no longer recommended in the PRISMA-DTA checklist [5]. Variability across studies was assumed, and exploration for potential causes of variability between image-based investigations using a priori subgroup analyses was planned. These included a number of study characteristics (year of publication, location, study design, reference standard, and duration between index test and reference standard), patient characteristics (percent male sex, mean age, total number of included patients, and total number of patients stratified by METAVIR grading), and imaging characteristics (VCTE/pSWE/2D-SWE: vendor used, technician experience, number of acquisitions, and threshold used; MRE: vendor used, MRI field strength, passive driver amplitude, segmentation method, and threshold used). Based on available data, completed subgroup analyses included location (east Asian country versus other countries), study design (prospective versus retrospective), number of centers (single-center versus multicenter), risk of bias (low risk of bias versus single domain with unclear or high risk of bias), vendor used, number of measurements for pSWE (<10 versus ≥ 10), MRI field strength (1.5 T versus 3 T), and MRI pulse sequence used (GRE versus SE-EPI). Analysis was performed using STATA 16 software (StataCorp, 2019, Stata Statistical Software: Release 15, College Station, TX, USA: StataCorp LP) and R software (R Core Team (2021, R: A language and environment for statistical computing; R Foundation for Statistical Computing, Vienna, Austria). 

## 3. Results

**Figure 1:**
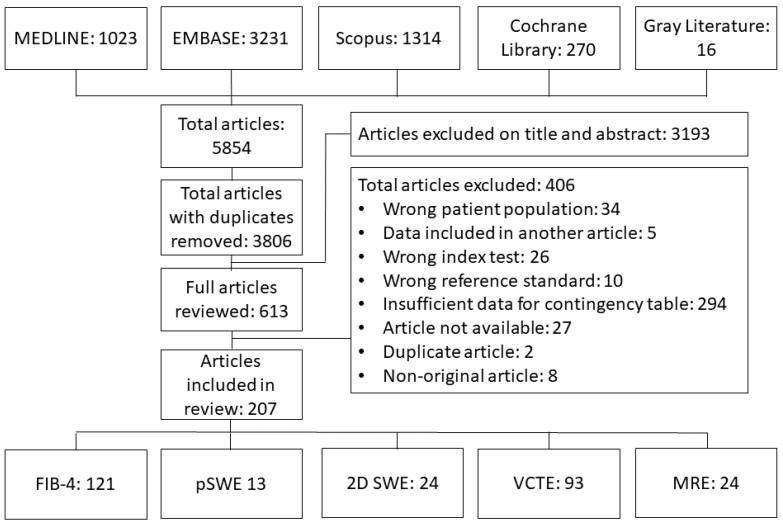
PRISMA flow diagram showing screening and selection of studies included in the systematic review.

The literature search PRISMA flow diagram is shown in Figure 1. Of 3806 articles reviewed, a total of 207 were included, with 121 articles evaluating FIB-4 (57,100 patients) [11,12,13,14,15,16,17,18,19,20,21,22,23,24,25,26,27,28,29,30,31,32,33,34,35,36,37,38,39,40,41,42,43,44,45,46,47,48,49,50,51,52,53,54,55,56,57,58,59,60,61,62,63,64,65,66,67,68,69,70,71,72,73,74,75,76,77,78,79,80,81,82,83,84,85,86,87,88,89,90,91,92,93,94,95,96,97,98,99,100,101,102,103,104,105,106,107,108,109,110,111,112,113,114,115,116,117,118,119,120,121,122,123,124,125,126,127,128,129,130,131], 13 articles evaluating pSWE (1033 patients) [14,132,133,134,135,136,137,138,139,140,141,142,143], 24 articles evaluating 2D-SWE (2592 patients) [11,13,116,140,144,145,146,147,148,149,150,151,152,153,154,155,156,157,158,159,160,161,162,163], 93 articles evaluating VCTE (24,628 patients) [12,15,19,21,22,23,24,26,27,28,29,30,31,32,33,34,35,36,37,39,40,41,42,43,44,45,46,47,48,49,67,116,133,134,136,137,138,140,142,146,150,151,152,154,159,160,162,163,164,165,166,167,168,169,170,171,172,173,174,175,176,177,178,179,180,181,182,183,184,185,186,187,188,189,190,191,192,193,194,195,196,197,198,199,200,201,202,203,204,205,206,207], and 24 articles evaluating MRE (3420 patients) [15,16,17,18,19,20,116,139,145,146,152,172,174,175,198,208,209,210,211,212,213,214,215,216]. Some articles included the evaluation of more than one modality.

### 3.1. Study, Patient, and Imaging Characteristics

Study and patient details for FIB-4 are shown in Appendix B. Included studies were performed across numerous countries using a mix of prospective and retrospective study designs. All studies used liver biopsy as the reference standard.

Study, patient, and imaging characteristics for studies evaluating pSWE, 2D-SWE, and VCTE are shown in Appendix C. Studies evaluating 2D-SWE used a mix of vendors including Canon, General Electric (GE), Siemens, SuperSonic Imagine, Toshiba, and Ultrasign. Studies evaluating pSWE used a mix of Siemens, Phillips, and Samsung. All VCTE studies used Echosens (FibroScan). Studies for each modality were performed internationally with a mix of prospective and retrospective study designs. Readers demonstrated varying degrees of experience with the technologies (reported as number of exams and/or years of experience). All studies used liver biopsy as the reference standard.

Study, patient, and imaging characteristics for studies evaluating MRE are shown in Appendix D. Most studies were performed in North America, with east Asian countries the next most common. A mix of magnet strengths (1.5 Tesla or 3 Tesla) and pulse sequences (2D gradient echo [2D-GRE] or spin echo echoplanar [SE-EPI] sequences) were used. Most studies used a 60 Hz passive driver and a manual region of interest. All studies used liver biopsy as the reference standard.

### 3.2. Diagnostic Accuracy by Modality

Pooled and weighted sensitivity and specificity values reported by threshold for each modality for detecting significant (METAVIR 0–1 versus 2–4) and advanced (METAVIR 0–2 versus 3–4) hepatic fibrosis are shown in Table 1. Specific evaluation of studies assessing FIB-4 thresholds of 1.3 and 2.67 are shown in Table 2. Sensitivities are 65% (95% CI 55–73%) and 72% (68–76%) for detecting significant and advanced hepatic fibrosis at a FIB-4 threshold of 1.3. Sensitivities are increased to 89% (84–93%) and 88% (85–91%) for excluding significant and advanced hepatic fibrosis at FIB-4 thresholds of 0.48 and 0.74, respectively. Specificities are 97% (94–99%) and 96% (94–97%) for detecting significant and advanced hepatic fibrosis at a FIB-4 threshold of 2.67 with sensitivities of 25% (18–33%) and 32% (27–38%), respectively. A lowered specificity of 88% (85–91%) for detecting advanced hepatic fibrosis is achieved with a threshold of ≥2.4 with a corresponding increased sensitivity of approximately 10% (42% [35–50%]).

Studies evaluating pSWE showed lower threshold values for achieving similar accuracies compared to 2D-SWE and VCTE. Thresholds with sensitivities nearing 90% for detecting significant and advanced hepatic fibrosis are 0.80 (88% [42–99%]) and 3.20 (88% [74–95%]), respectively, for pSWE, 5.10 (88% [78–94%]) and 4.92 (88% [70–94%]) for 2D-SWE, and 6.10 (88% [92–92%]) and 7.18 (88% [85–91%]) for VCTE. Sensitivities of 90% (80–95%) and 91% (84–95%) for detecting significant and advanced hepatic fibrosis are achieved with MRE thresholds of 2.32 and 1.24, respectively.

Evaluations of vendor-specific thresholds and accuracies are shown in Table 3. Accuracies of most vendor-specific thresholds for pSWE, 2D-SWE, and MRE could not be performed due to the limited number of individual studies and/or contingency tables available by fibrosis severity. More than one vendor comparison was only available for 2D-SWE (GE and Supersonic Imagine), with GE demonstrating higher thresholds for similar accuracies. For example, sensitivities of 88% (81–96) and 88% (39–99%) for detecting advanced hepatic fibrosis were achieved with thresholds of 6.1 for GE and 3.3 for Supersonic Imagine, respectively.

### 3.3. Subgroup Analysis

Subgroup analyses evaluating reported thresholds of 7–9 kPa (8 ± 1 kPa) for pSWE, 2D-SWE, VCTE, and MRE are shown in Table 4. No significant differences were identified between sensitivities across all modalities and fibrosis severity. Studies using more than 10 measurements with pSWE had a higher specificity for detecting advanced hepatic fibrosis (84% vs. 57%, *p* < 0.05). GE demonstrated a higher specificity than Supersonic Imagine for detecting advanced hepatic fibrosis using 2D-SWE (86% vs. 62%, *p* < 0.05) but no difference was seen for Canon or Siemens. Studies with a single domain rated as unclear or high risk of bias demonstrated a higher specificity than low-risk studies for detecting advanced hepatic fibrosis using VCTE (79% vs. 61%, *p* < 0.05). No other differences were seen between specificities across modalities and fibrosis severity.

### 3.4. Risk of Bias Assessment

Risk of bias and applicability concerns are shown for each study separated by modality in Appendix E. Most imaging-based modalities (pSWE, 2D-SWE, VCTE, and MRE) were deemed high risk of bias for the index test as most studies used a retrospective threshold to evaluate accuracy. Many studies would also report a 90% sensitivity and/or 90% specificity, and these were included for multilevel evaluation when reported. Most studies evaluating imaging-based modalities were deemed low or unclear risk for patient selection, index test, and flow and timing. Most studies evaluating FIB-4 were deemed low or unclear risk of bias across all four domains.

## 4. Discussion

The European Association for the Study of Liver (EASL), American Association for the Study of Liver Disease (AASLD), and the American Gastroenterological Association (AGA) have each recently published guidelines for population-level screening of patients with suspected or diagnosed MASLD [1,2,3]. These guidelines use a combination of blood-based, then imaging-based recommendations. An initial blood-based investigation is suggested to be a more cost-effective first step with each guideline recommending the use of FIB-4 as the investigation of choice. In these pathways, a FIB-4 score of <1.3 characterizes a patient as low risk for liver-related outcomes, while a FIB-4 score of >2.67 characterizes a patient as high risk. Scores of 1.3–2.67 are deemed to be indeterminate and warrant further evaluation with an imaging-based investigation. Our meta-analysis of over 50,000 international patients receiving FIB-4 testing suggests that a “rule out” threshold of 1.3 results in sensitivities of only 71–74% for significant and advanced hepatic fibrosis. By contrast, a threshold of <0.75 can result in a sensitivity of nearly 90% for excluding advanced hepatic fibrosis. Conversely, a “rule in” threshold warranting referral to hepatology of 2.67 can result in specificities of 96–97% for detecting significant and advanced hepatic steatosis but with a sensitivity of only 32% (95% CI 27–38%). A slightly lower “rule in” threshold of ≥2.4 would result in a specificity nearing 90% and could also increase sensitivity by approximately 10% (42% [35–50%]), but it may result in an increase of too many patients without hepatic fibrosis being referred to hepatology services.

For imaging-based investigations recommended in indeterminate scenarios, existing guidelines recommend a modality- and vendor-agnostic threshold of 8 kPa as the low-risk threshold with two guidelines recommending follow-up with FIB-4 in 1–3 years [1,3] and one guideline recommending either follow-up or liver biopsy in these low-risk patients [2]. However, our meta-analysis indicates that a threshold of <8 kPa is likely to have a sensitivity between 70–80% when using 2D-SWE or VCTE for detecting advanced hepatic fibrosis, and a lower sensitivity when using pSWE. By contrast, individualized sensitivities of nearly 90% can be achieved with independent modality thresholds of 3 kPa for pSWE, 5 kPa for 2D SWE, and 7 kPa for VCTE (“3/5/7 rule”) for detecting advanced hepatic fibrosis. Conversely, a “rule in” threshold of 13 kPa could result in specificities of nearly 90% detecting advanced hepatic fibrosis with pSWE and VCTE and significant hepatic fibrosis with 2D-SWE. These thresholds would more closely align with the comparatively more conservative thresholds recommended by the Society of Radiologists in Ultrasound (SRU) for pSWE and 2D-SWE, which use a “rule out” threshold of 5 kPa and an indeterminate range of 5–13 kPa [4].

Finally, the ASSLD guideline recommends an MRE threshold of ≤3 kPa to risk stratify indeterminate risk patients into a low-risk category [2]. Our meta-analysis suggests that a threshold of 3 kPa would result in a sensitivity of around 80% for detecting significant and advanced hepatic fibrosis, while a threshold closer to 2.5 kPa would increase the sensitivity to around 90% for detecting significant hepatic fibrosis. A lower threshold was identified in our meta-analysis for detecting advanced hepatic fibrosis, which is felt to be a function of limited underlying studies evaluating this disease severity, so preference is placed on the identified threshold for significant hepatic fibrosis.

**Figure 2:**
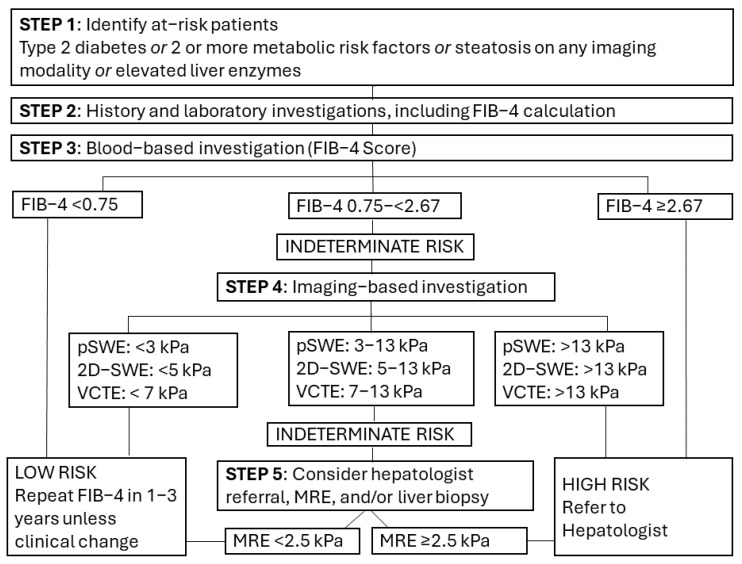
Proposed two-tier blood-based followed by image-based risk stratification algorithm for population screening of patients with suspected or diagnosed MASLD. Metabolic risk factors = central obesity, high triglycerides, low HDL cholesterol, hypertension, prediabetes, or insulin resistance.

Based on these results, a revised proposed risk stratification pathway is shown in Figure 2. This proposed pathway lowers the “rule out” FIB-4 threshold from existing guidelines and provides modality-specific thresholds for imaging-based investigations. While this is likely to result in higher frequency use of imaging-based investigations and possibly a higher number of hepatology referrals for “indeterminate risk” patients, the ability to risk stratify MASLD patients into a “low-risk” category would be improved with the sensitivity increasing from below 80% to approximately 90%.

### 4.1. Limitations

Our meta-analysis is limited by near-universal issues of presumed variability in diagnostic test accuracy systematic reviews [5]. In an effort to identify potential sources of variability within different modalities, an a priori subgroup analysis explored several potential causes for variability separated by modality and fibrosis severity with thresholds between 7 and 9 kPa. No significant differences in sensitivity were identified in these thresholds, and only three differences in specificity were identified (higher specificities identified with ≥10 measurements for pSWE, for GE versus Supersonic Imagine vendors for 2D-SWE, and for studies with unclear and/or high-risk domains for VCTE). This likely under-recognizes other potential sources for variability which were not explored due to lack of available data or non-extraction. One area of particular concern was vendor-specific accuracies for pSWE, 2D-SWE, and MRE as these technologies rely on vendor-specific proprietary software parameters. Unlike VCTE, which uses a single-vendor product, these modalities may be dependent on the vendor-specific acquisition. However, subgroup analysis identified only a statistically lower accuracy for Supersonic Imagine compared to GE for 2D-SWE with thresholds between 7 and 9 kPa for detecting advanced but not significant hepatic fibrosis. A separate vendor-specific analysis of all thresholds was also performed to determine the appropriateness of vendor-specific threshold recommendations, although limited available primary data precluded detailed comparison between these vendors stratified by modality and fibrosis severity. The available data suggest that Supersonic Imagine may benefit from a lower “rule out” threshold recommendation than GE to sustain sensitivities around 90% for 2D-SWE, although this was not statistically significant. Another potential area for variability between modalities was the conversions needed for some studies such as shear wave velocities to Young’s modulus (most often needed in pSWE studies) and conversion to a METAVIR grading system from other histologic grading systems. It is possible that differences between vendor-specific conversion calculations and assumptions (such as density of soft tissue and liver) may have contributed to lower threshold values for pSWE compared to 2D-SWE and VCTE, for example. As such, it is recommended that manual conversions from shear wave speed to Young’s modulus should utilize the same calculation as was used in this study when referencing the modified threshold recommendations outlined here. Additionally, to reduce the risk for potential variability within an individual, surveillance of the same patient would best be performed using the same modality and, ideally, the same machine as was used previously. The SRU suggests a variance of 10% using the same machine to indicate a clinically significant change in individualized surveillance for pSWE and 2D-SWE [4].

### 4.2. Future Research

Several avenues of future research are needed. First, continued accuracy studies, particularly for image-based investigations including pSWE, 2D-SWE, and MRE will help future data synthesis identify important subgroups to further refine threshold recommendations. In particular, further studies evaluating vendor-specific accuracies and thresholds will help define the potential utility of vendor-specific threshold recommendations. Additionally, as population screening gains traction on a national and international level, large-scale longitudinal evaluation of the change in referral patterns and patient outcomes will be required. Through this process, optimal surveillance recommendations—currently based on expert consensus and dependent on individual guidelines and regional practice—can be further refined. For those receiving imaging-based follow-up, evaluation of individual modality-specific and clinically significant thresholds for change are needed. This includes studies evaluating specific subgroups of MASLD which can inform referral pathways and threshold recommendations. For example, limited literature evaluating patients <18 years of age is available, and recommended guidelines for the pediatric population with MASLD are not clearly established. Alternatively, type 2 diabetes is known to be the strongest predictor for developing MASLD/MASH-related advanced fibrosis and liver related complications and may warrant shorter surveillance periods [1]. Additionally, a cost-analysis study to determine the economic consequences of the proposed changes outlined in this study can help inform economic decisions necessary for these algorithmic changes.

### 4.3. Conclusions

In summary, this comprehensive systematic review, including more than 200 studies and over 80,000 index tests, offers novel insights into the accuracies of individual blood-based and imaging-based guidelines available to date. Our study suggests that existing guideline thresholds for determining low-risk patients with FIB-4 should be lowered, and image-based investigations for indeterminate patients should be individualized by modality. A revised proposed algorithm has been provided which can be used to inform future guideline development and revisions, and, more importantly, population-based screening and risk stratification practices.

## Figures and Tables

**Table 1:** Pooled and weighted sensitivity and specificity values reported by threshold for FIB-4, pSWE, 2D-SWE, VCTE, and MRE for detecting significant and advanced hepatic fibrosis in MASLD patients. Weighted sensitivities to specificities included 0.3:0.7, 0.5:0.5, and 0.7:0.3.

		Sensitivity Weight 0.3			Sensitivity Weight 0.5			Sensitivity Weight 0.7		
Modality	Fibrosis Severity	Threshold	Sensitivity [%, 95% CI]	Specificity [%, 95% CI]	Threshold	Sensitivity [%, 95% CI]	Specificity [%, 95% CI]	Threshold	Sensitivity [%, 95% CI]	Specificity [%, 95% CI]
FIB-4	Significant	2.34	36 [27–47]	89 [84–93]	1.06	68 [61–75]	68 [61–75]	0.48	89 [84–93]	36 [27–47]
	Advanced	2.39	42 [35–50]	88 [85–91]	1.33	70 [63–76]	70 [65–75]	0.74	88 [85–91]	42 [35–50]
pSWE	Significant	35.90	52 [10–92]	88 [42–99]	5.37	74 [60–84]	74 [59–84]	0.80	88 [42–99]	52 [10–92]
	Advanced	12.97	61 [33–83]	88 [74–95]	6.43	78 [60–89]	78 [65–87]	3.20	88 [74–95]	61 [33–83]
2D-SWE	Significant	13.59	54 [35–72]	88 [78–94]	8.31	74 [60–85]	74 [62–84]	5.10	88 [78–94]	54 [35–72]
	Advanced	18.63	60 [38–79]	88 [79–94]	9.58	77 [61–88]	77 [67–85]	4.92	88 [79–94]	60 [38–79]
VCTE	Significant	11.88	48 [37–60]	88 [82–92]	8.48	72 [62–80]	72 [63–79]	6.10	88 [82–92]	48 [37–60]
	Advanced	13.21	56 [48–64]	88 [85–91]	9.74	75 [69–81]	75 [70–80]	7.18	88 [85–91]	56 [48–64]
MRE	Significant	3.70	69 [50–83]	90 [80–95]	2.93	82 [67–90]	82 [68–90]	2.32	90 [80–95]	69 [50–83]
	Advanced	5.98	75 [61–85]	91 [84–95]	2.73	85 [75–91]	85 [76–90]	1.24	91 [84–95]	75 [61–85]

Significant hepatic fibrosis = METAVIR 0–1 versus METAVIR 2–4; advanced hepatic fibrosis = METAVIR 0–2 versus METAVIR 3–4; FIB-4 = Fibrosis 4; pSWE = point shear wave elastography; 2D-SWE = 2D shear wave elastography; VCTE = vibration-controlled elastography; and MRE = magnetic resonance elastography.

**Table 2:** Pooled sensitivity and specificity values for FIB-4 thresholds of 1.3 and 2.67 for detecting significant and advanced hepatic fibrosis in MASLD patients.

Hepatic Fibrosis Severity	FIB–4 Threshold	No. Studies	No. Patients	Sensitivity [% (95% CI)]	Specificity [% (95% CI)]
Significant	1.3	13	7975	65 (55–73)	74 (66–81)
Advanced	1.3	44	21,409	72 (68–76)	71 (66–75)
Significant	2.67	7	3741	25 (18–33)	97 (94–99)
Advanced	2.67	50	29,953	32 (27–38)	96 (94–97)

Significant hepatic fibrosis = METAVIR 0–1 versus METAVIR 2–4; advanced hepatic fibrosis = METAVIR 0–2 versus METAVIR 3–4.

**Table 3:** Vendor-specific pooled and weighted sensitivity and specificity values reported by threshold for pSWE, 2D-SWE, and MRE for detecting significant and advanced hepatic fibrosis in MASLD patients. Weighted sensitivities to specificities included 0.3:0.7, 0.5:0.5, and 0.7:0.3.

			No. Studies	No. Contigency Tables	Sensitivity Weight 0.3			Sensitivity Weight 0.5			Sensitivity Weight 0.7		
Modality	Fibrosis Severity	Vendor			Threshold	Sensitivity [%, 95% CI]	Specificity [%, 95% CI]	Threshold	Sensitivity [%, 95% CI]	Specificity [%, 95% CI]	Threshold	Sensitivity [%, 95% CI]	Specificity [%, 95% CI]
2D-SWE	Significant	Canon	4	6	NA	NA	NA	NA	NA	NA	NA	NA	NA
		GE	7	11	14.5	49 (12–87)	91 (0–100)	9	73 (47–89)	73 (51–87)	5.6	88 (72–95)	49 (30–68)
		Siemens	2	4	NA	NA	NA	NA	NA	NA	NA	NA	NA
		Supersonic Imagine	8	10	13.6	52 (23–80)	88 (80–96)	8.2	74 (55–87)	74 (57–86)	5	88 (63–97)	52 (23–80)
	Advanced	Canon	4	5	NA	NA	NA	NA	NA	NA	NA	NA	NA
		GE	7	10	12.3	59 (28–84)	88 (70–96)	8.7	77 (54–90)	77 (61–87)	6.1	88 (71–96)	59 (37–77)
		Siemens	2	4	NA	NA	NA	NA	NA	NA	NA	NA	NA
		Supersonic Imagine	9	12	35	58 (10–95)	88 (36–99)	10.8	76 (58–88)	76 (64–85)	3.3	88 (39–99)	58 (12–93)
pSWE	Significant	Siemens	5	8	15.1	54 (61–96)	88 (29–99)	4.4	75 (62–84)	75 (61–84)	1.3	88 (37–99)	54 (8–94)
		Phillips	5	5	NA	NA	NA	NA	NA	NA	NA	NA	NA
		Samsung	1	3	NA	NA	NA	NA	NA	NA	NA	NA	NA
	Advanced	Siemens	7	10	8.8	67 (41–86)	89 (77–96)	5.9	81 (64–91)	81 (69–89)	4	89 (78–95)	67 (52–79)
		Phillips	5	5	NA	NA	NA	NA	NA	NA	NA	NA	NA
		Samsung	1	3	NA	NA	NA	NA	NA	NA	NA	NA	NA
MRE	Significant	GE	13	19	3.9	70 (52–83)	80 (81–95)	3	82 (67–91)	82 (69–90)	2.4	90 (72–97)	70 (41–88)
		Siemens	2	2	NA	NA	NA	NA	NA	NA	NA	NA	NA
	Advanced	GE	10	14	5.9	75 (57–88)	91 (83–96)	2.6	85 (73–92)	85 (76–91)	1.1	91 (72–98)	75 (45–92)
		Siemens	1	1	NA	NA	NA	NA	NA	NA	NA	NA	NA
		Phillips	1	1	NA	NA	NA	NA	NA	NA	NA	NA	NA

Significant hepatic fibrosis = METAVIR 0–1 versus METAVIR 2–4; advanced hepatic fibrosis = METAVIR 0–2 versus METAVIR 3–4; No. studies = number of studies; No. contingency tables = number of contingency tables (which may include >1 table per study if multiple thresholds are reported); NA = not analyzable.

**Table 4:** Pooled sensitivity and specificity subgroup analyses evaluating reported thresholds of 7–9 kPa (8 ± 1 kPa) for pSWE, 2D-SWE, VCTE, and MRE for detecting significant and advanced hepatic fibrosis in MASLD patients.

Modality	Fibrosis Severity	Subgroup	No. Studies	No. Patients	Sensitivity, % (95% CI)	Specificity, % (95% CI)	Significance
pSWE	Significant	East Asian Countries	0	0	N/A	N/A	N/A
		Other Countries	3	232	78 (70–84)	80 (71–87)	
		Vendor: Siemens	1	27	N/A	N/A	N/A
		Vendor: Philips	1	159	N/A	N/A	
		Vendor: Samsung	1	46	N/A	N/A	
		<10 measurements	2	92	72 (59–83)	68 (52–81)	NS
		≥10 measurements	3	232	77 (69–93)	80 (71–87)	
		RoB: Low	3	138	73 (62–81)	70 (57–81)	NS
		RoB: Unclear and/or High	2	186	78 (69–85)	82 (72–89)	
	Advanced	East Asian Countries	2	117	75 (53–88)	62 (40–80)	NS
		Other Countries	5	518	72 (62–81)	82 (74–89)	
		Vendor: Siemens	2	263	62 (51–71)	86 (67–95)	NS
		Vendor: Philips	4	326	80 (70–87)	77 (61–88)	
		Vendor: Samsung	1	46	67 (41–87)	71 (51–87)	
		<10 measurements	2	146	71 (52–85)	57 (41–72)	*p* < 0.05
		≥10 measurements	4	472	73 (60–83)	84 (76–89)	
		RoB: Low	3	299	73 (62–81)	70 (57–81)	NS
		RoB: Unclear and/or High	4	336	78 (69–85)	82 (72–89)	
2D–SWE	Significant	East Asian Countries	9	1005	84 (72–91)	85 (77–91)	NS
		Other Countries	6	649	83 (74–89)	83 (75–88)	
		Prospective Study Design	12	1360	84 (74–90)	82 (73–89)	NS
		Retrospective Study Design	4	450	84 (76–90)	81 (73–87)	
		Vendor: Canon	3	339	92 (75–97)	87 (73–94)	NS
		Vendor: GE	5	518	82 (66–91)	81 (63–91)	
		Vendor: Siemens	2	301	85 (64–94)	74 (51–88)	
		Vendor: Supersonic Imagine	6	652	83 (70–91)	79 (64–89)	
		RoB: Low	9	1216	85 (74–92)	75 (63–84)	NS
		RoB: Unclear and/or High	7	594	84 (76–90)	81 (73–87)	
	Advanced	East Asian Countries	10	1199	86 (78–92)	81 (75–86)	NS
		Other Countries	5	478	87 (74–94)	83 (74–90)	
		Prospective Study Design	12	1260	86 (80–91)	84 (78–89)	NS
		Retrospective Study Design	2	223	86 (68–95)	82 (76–86)	
		Vendor: Canon	3	339	98 (84–100)	82 (72–98)	*p* < 0.05
		Vendor: GE	7	668	81 (73–87)	86 (78–91)	
		Vendor: Siemens	1	222	91 (84–96)	76 (68–83)	
		Vendor: Supersonic Imagine	2	230	90 (77–96)	62 (45–76)	
		RoB: Low	7	860	86 (77–92)	83 (77–90)	NS
		RoB: Unclear and/or High	7	623	87 (76–93)	81 (69–88)	
VCTE	Significant	East Asian Countries	12	1774	80 (71–86)	74 (63–82)	NS
		Other Countries	23	5065	80 (74–85)	66 (58–73)	
		Prospective Study Design	28	5010	79 (75–83)	72 (66–78)	NS
		Retrospective Study Design	9	2380	84 (79–89)	59 (50–69)	
		Single Hospital Design	22	2820	77 (72–82)	70 (64–77)	NS
		Multicenter Design	18	5122	81 (76–85)	69 (61–75)	
		RoB: Low	23	2937	77 (72–82)	72 (64–78)	NS
		RoB: Unclear and/or High	17	4773	82 (76–86)	65 (56–73)	
	Advanced	East Asian Countries	14	2178	87 (80–92)	71 (59–81)	NS
		Other Countries	25	8691	87 (83–91)	69 (60–77)	
		Prospective Study Design	29	7541	88 (84–91)	72 (66–78)	NS
		Retrospective Study Design	12	5328	87 (82–91)	64 (55–71)	
		Single Hospital Design	22	3148	84 (79–89)	71 (61–79)	NS
		Multicenter Design	22	9881	89 (86–92)	70 (60–78)	
		RoB: Low	22	7065	88 (84–91)	61 (52–69)	*p* < 0.05
		RoB: Unclear and/or High	19	5804	87 (82–90)	79 (72–84)	
MRE	Significant	East Asian Countries	7	1125	88 (82–92)	84 (77–89)	NS
		Other Countries	10	970	77 (67–84)	88 (83–91)	
		Prospective Study Design	10	1277	83 (73–90)	87 (81–91)	NS
		Retrospective Study Design	3	394	88 (73–95)	83 (71–91)	
		Vendor: GE	9	1276	86 (75–92)	84 (78–89)	NS
		Vendor: Siemens	2	166	85 (59–96)	93 (80–98)	
		Magnet Strength: 1.5T	4	389	84 (69–93)	82 (71–90)	NS
		Magnet Strength: 3T	8	1112	83 (71–91)	87 (80–91)	
		Pulse Sequence: GRE	7	642	84 (72–91)	82 (72–88)	NS
		Pulse Sequence: SE–EPI	4	458	83 (66–93)	89 (77–95)	
		RoB: Low	10	1241	82 (72–89)	87 (81–91)	NS
		RoB: Unclear and/or High	4	498	83 (71–91)	86 (78–92)	
	Advanced	East Asian Countries	1	201	82 (73–89)	92 (84–96)	NS
		Other Countries	4	305	86 (75–93)	85 (80–89)	
		Prospective Study Design	5	403	86 (76–93)	88 (83–92)	N/A
		Retrospective Study Design	0	0	N/A	N/A	
		Vendor: GE	3	292	82 (68–91)	91 (80–96)	N/A
		Vendor: Siemens	0	0	N/A	N/A	
		Magnet Strength: 1.5T	1	59	92 (73–99)	89 (73–97)	NS
		Magnet Strength: 3T	3	292	82 (68–91)	90 (81–95)	
		Pulse Sequence: GRE	3	253	85 (73–92)	86 (80–91)	NS
		Pulse Sequence: SE–EPI	1	98	87 (60–98)	96 (90–99)	
		RoB: Low	3	292	82 (68–91)	91 (82–96)	NS
		RoB: Unclear and/or High	2	111	93 (74–98)	81 (62–92)	

Subgroup analysis performed by modality and fibrosis severity for studies reporting thresholds between 7–9 kPa (8 ± 1 kPa). Categories with an insufficient number of studies could not be analyzed and are labeled as “N/A”; No. studies = number of studies; No. patients = number of patients included in subgroup assessment; GE = General Electric; RoB = Risk of Bias stratified by all domains that are deemed low risk versus studies with any single domain deemed unclear or high risk of bias; GRE = gradient echo; SE-EPI = spin echoplanar; N/A = not analyzable; NS = not significant.

## Supplementary Materials

The following supporting information can be downloaded at: https://www.mdpi.com/article/10.3390/diagnostics15131598/s1, Table S1: PRISMA checklist.

## Abbreviations

CLD = Chronic liver disease; MASH = metabolic dysfunction-associated steatohepatitis; MASLD = Metabolic dysfunction-associated steatotic liver disease; MRE = Magnetic resonance elastography; NAFLD = Non-alcoholic fatty liver disease; PRISMA-DTA = Preferred Reporting Items for Systematic Reviews and Meta-analysis–Diagnostic Test Accuracy; SE-EPI = spin echo–echoplanar imaging; SWE = Shear wave elastography; QUADAS 2 = Quality Assessment of Diagnostic Accuracy Studies-2; VCTE = Vibration controlled transient elastography

## Appendix A

**Table A1:** Search strategy by database.

Database	Search Strategy
MEDLINE	(exp Non-alcoholic Fatty Liver Disease/OR nonalcoholic fatty liver disease.mp. OR NAFLD.mp. OR metabolic dysfunction-associated steatotic liver disease.mp. OR MASLD.mp.) AND (FIB-4.mp. OR exp Elasticity Imaging Techniques/OR [transient elastography.mp. OR Fibroscan.mp.] OR shear wave elastography.mp. OR SWE.mp. OR pSWE.mp. OR 2D-SWE.mp. OR magnetic resonance elastography.mp. OR MRE.mp.) AND (exp “Sensitivity and Specificity”/OR sensitivity.mp. OR specificity.mp. OR performance.mp. OR exp Data Accuracy/OR accuracy.mp.)
EMBASE	(exp nonalcoholic fatty liver/OR nonalcoholic fatty liver disease.mp. OR NAFLD.mp. OR Metabolic dysfunction-associated steatotic liver disease.mp. OR MASLD.mp.) AND (FIB-4.mp. OR exp elastography/OR exp transient elastography/or exp elasticity/OR transient elastography.mp. OR Fibroscan.mp. OR exp elastograph/OR exp shear wave elastography/OR shear wave elastography.mp. OR SWE.mp. OR pSWE.mp. OR 2D-SWE.mp. OR exp. magnetic resonance elastography/OR magnetic resonance elastography.mp. OR MRE.mp.) AND (exp “sensitivity and specificity”/OR sensitivity.mp. OR specificity.mp. OR performance.mp. OR exp performance/OR exp data accuracy/OR exp diagnostic test accuracy study/OR acciracy.mp. OR exp diagnostic accuracy/)
Scopus	(TITLE-ABS-KEY ((non-alcoholic AND fatty AND liver AND disease) OR nafld OR (metabolic AND dysfunction-associated AND steatotic AND liver AND disease) OR masld) AND TITLE-ABS-KEY (fib-4 OR (transient AND elastography) OR fibroscan OR (shear AND wave AND elastography) OR swe OR pswe OR 2d-swe OR (magneticAND resonance AND elastography) OR mre) AND TITLE-ABS-KEY (sensitivity OR specificity OR performance OR accuracy))
Cochrane Library	(nonalcoholic fatty liver disease OR nafld OR metabolic dysfunction-associated steatotic liver disease OR masld) AND (FIB-4 OR transient elastography OR fibroscan OR shear wave elastography OR see OR pswe OR 2d-swe OR magnetic resonance elastography OR mre) AND (sensitivity OR specificity OR performance OR accuracy)

Database search was performed on 25 March 2024.

## Appendix B

**Table A2:** Study, patient, and imaging characteristics of included studies evaluating the accuracy of FIB-4 for detecting significant and/or advanced hepatic fibrosis in MASLD patients.

Author	Year	Country	Study Design	Centers	Total # Patients	Mean Age (Range)	Male Sex (%)	Total Patients
Chen [11]	2022	China	Retrospective	Single-center	100	32 (16–65)	31	100
Lee [12]	2022	Republic of Korea	Retrospective	Multicenter	251	44 (34–56)	53	251
Takeuchi [13]	2018	Japan	Prospective	Single-center	71	51 (18–82)	65	71
da Silva [14]	2021	Brazil	Prospective	Single-center	108	45	21	108
Duman [15]	2024	Turkey	Retrospective	Single-center	119	54 (20–73)	32	119
Cui [16]	2015	USA	Prospective	Single-center	102	51	41	102
Inada [17]	2022	Japan	Retrospective	Single-center	105	65 (58–72)	45	105
Tamaki [18]	2023	USA and Japan	Both	Multicenter	806	NR	48	806
Ogawa [19]	2018	Japan	Retrospective	Single-center	165	54	58	165
Jung [20]	2021	USA	Prospective	Multi-center	238	51 (37–65)	46	238
Armandi [21]	2023	Italy	Prospective	Single-center	96	50 (20–74)	62	96
Wong [22]	2010	France and HK	Prospective	Multicenter	245	51	55	245
Boursier [23]	2016	France	Prospective	Multicenter	588	56	57	452
Pennisi [24]	2023	Italy	Prospective	Single-center	520	52 (25–78)	65	520
Pennisi [25]	2023	Multi-country	Retrospective	Multicenter	1780	61 (54–67)	58	1780
Prat [26]	2019	United Kingdom	Retrospective	Single-center	27	48 (38–58)	100	27
Arvaniti [27]	2023	Greece	Retrospective	Single-center	38	50 (16–69)	61	38
Kao [28]	2020	Taiwan	Prospective	Single-center	123	36	29	123
Castera [29]	2023	France	Prospective	Multicenter	163	59 (median)	58	163
Petta [30]	2019	Italy, France, Hong Kong, China	Prospective	Multicenter	968	50	63	968
Staufer [31]	2019	Austria	Prospective	Multicenter	186	52 (39–60)	57	186
Boursier [32]	2023	France	Prospective	Multicenter	1051	58 [50–66]	60	1051
Noureddin [33]	2023	USA	Retrospective	Multicenter	548	58	35	548
Noureddin [34]	2021	USA	Retrospective	Multicenter	1308	57 (median)	NR	1308
Cheung [35]	2023	Malaysia, Hong Kong, China	Prospective	Multicenter	431	48	57	431
Bhadoria [36]	2017	India	Retrospective	Single-center	779	44	75	779
Anstee [37]	2019	Multicenter	Prospective	Multicenter	3123	59	58	3123
Anstee [38]	2020	UK	Prospective	Multicenter	420	58 (44–74)	52	420
Labenz [39]	2018	Germany	Prospective	Single-center	243	51 (19–93)	53	243
Arora [40]	2023	India, Singapore	Retrospective	Multicenter	641	43	55	641
Barritt [41]	2019	USA	Retrospective	Multicenter	1549	59	45	1549
Harrison [42]	2020	USA	Prospective	Multi-center	320	55	38	307
Petta [43]	2015	Italy	Prospective	Single-center	179	45 (18–72)	68	179
Gabriel-Medina [44]	2023	Spain	Prospective	Single-center	140	59	42	140
Zhang [45]	2023	China	Prospective	Single-center	71	46	68	71
Eddowes [46]	2019	UK	Prospective	Multicenter	356	53 (42–64)	57	356
Sanyal [47]	2023	Multiple	Not specified	Multicenter	1434	55	51	3176
Boursier [48]	2019	France	Prospective	Multicenter	938	57 (18–80)	59	938
Bertot [49]	2023	Australia	Retrospective	Single-center	271	52 (40–64)	40	271
Kobayashi [50]	2017	Japan	Retrospective	Single-center	229	56 (45–64)	46	229
Eren [51]	2022	Turkey	Retrospective	Multicenter	560	48 (18–71)	53	560
Singh [52]	2020	USA	Retrospective	Single-center	1157	51	35	1157
Marella [53]	2020	USA	Retrospective	Single-center	907	47	68	907
Treeprasertsuk [54]	2016	Thailand	Prospective	Single-center	139	41	47	139
McPherson [55]	2013	UK	Retrospective	Single-center	305	48	63	305
Kolhe [56]	2019	India	Retrospective	Single-center	100	46 (18–80)	53	100
Kaya [57]	2019	Turkey	Retrospective	Single-center	463	46	53	463
Nones [58]	2017	Brazil	Retrospective	Multicenter	67	55	37	67
Balakrishnan [59]	2018	USA	Cross-sectional	Single-center	122	47	20	122
Alkayyali [60]	2020	Turkey	Retrospective	Single-center	349	48 (38–58)	43	349
Nielsen [61]	2021	Denmark	Retrospective	Multicenter	517	55 (54–56)	52	517
Ampuero [62]	2020	Spain, France, Italy, Cuba, China	Retrospective	Multicenter	2452	52 (18–57)	55	2452
Sang [63]	2021	China, Malaysia, India	Retrospective	Multicenter	540	47 (18–57)	52	540
Shima [64]	2020	Japan	Retrospective	Single-center	278	58 (18–57)	48	278
Seko [65]	2023	Japan	Retrospective	Multicenter	371	61 (17–85)	43	371
Siddiqui [66]	2019	USA	Retrospective	Multicenter	1904	50 (18–57)	37	1904
Li [67]	2024	Hong Kong	Retrospective	Single-center	279	52 (18–57)	55	599
Sanyal [68]	2023	USA	Retrospective	Multicenter	1073	53 (10–56)	38	1073
Moon [69]	2023	Republic of Korea	Retrospective	Single-center	118	NR	NR	118
Zambrano-Huailla [70]	2020	Peru, Brazil, Argentina	Retrospective	Multicenter	379	46 (18–75)	30	379
Yang [71]	2022	China	Retrospective	Single-center	309	46 (18–57)	52	309
Giammarino [72]	2022	USA	Retrospective	Single-center	244	NR	NR	244
Nishikawa [73]	2016	Japan	Retrospective	Single-center	134	52 (18–57)	49	134
Kakisaka [74]	2018	Japan	Retrospective	Single-center	125	51	46	125
Schmitz [75]	2020	Germany	Prospective	Single-center	141	43	27	141
Zhang [76]	2023	China	Retrospective	Single-center	105	46 (15–69)	52	105
Balakrishnan [77]	2021	USA	Retrospective	Single-center	99	47	74	99
Kariyama [78]	2022	Japan	Retrospective	Multicenter	1059	55 (14–87)	52	1059
Mohammed [79]	2019	Egypt	Prospective	Single-center	100	47	38	100
de la Tijera [80]	2021	Mexico	Retrospective	Multicenter	222	46 (37–54)	26	222
McPherson [81]	2015	UK	Prospective	Multicenter	634	69 (66–72)	35	634
Shah [82]	2020	USA	Prospective	Multicenter	2056	NR	NR	2056
Maurice [83]	2021	UK, USA, Italy, Canada	Retrospective	Multicenter	116	48	93	116
Zhou [84]	2019	China	Prospective	Single-center	207	42 (18–75)	73	207
Kouvari [85]	2023	USA, Italy, Greece, Australia	Prospective	Multicenter	455	53 (51–56)	52	455
Ballestri [86]	2021	Italy	Prospective	Single-center	107	48	72	107
Noureddin [87]	2022	USA	Retrospective	Multicenter	232	56	60	232
Singh [88]	2022	India	Prospective	Single-center	129	40	NR	129
Prasad [89]	2020	India	Retrospective	Single-center	240	39	78	240
Kim [90]	2022	USA	Retrospective	Single-center	363	51 (median)	46	363
Yoneda [91]	2013	Japan	Retrospective	Multicenter	1102	60	NR	1102
Qadri [92]	2022	Finland	Retrospective	Single-center	378	50 (18–75)	29	378
Miller [93]	2019	USA	Retrospective	Single-center	354	50 (37–63)	42	354
Drolz [94]	2021	Germany	Retrospective	Single-center	368	47 (35–56)	43	368
Meneses [95]	2020	Spain	Prospective	Single-center	50	49 (18–57)	30	50
Alqahtani [96]	2021	USA	Retrospective	Single-center	584	43 (18–57)	21	584
De Carli [97]	2020	Brazil	Retrospective	Single-center	323	37	76	266
Ito [98]	2023	Japan, Taiwan, Korea	Retrospective	Multicenter	1489	46 (18–57)	54	1489
Bril [99]	2020	USA	Retrospective	Single-center	213	58 (50–66)	84	213
Satapathy [100]	2019	USA	Retrospective	Multicenter	269	NR	NR	269
Moon [101]	2024	Republic of Korea	Retrospective	Multicenter	231	46 (18–57)	54	231
Schwenger [102]	2023	Canada	Retrospective	Single-center	170	47 (18–57)	79	131
Andrade [103]	2022	Brazil	Retrospective	Single-center	143	48 (19–68)	34	143
Aida [104]	2015	Japan	Retrospective	Single-center	148	61 (46–70)	36	148
Huang [105]	2023	China	Prospective	Single-center	373	31 (18–57)	34	373
McPherson [106]	2010	UK	Retrospective	Single-center	145	51 (18–57)	61	145
Kaya [107]	2020	Turkey	Retrospective	Single-center	463	46	48	463
Xun [108]	2012	China	Retrospective	Single-center	152	37 (18–57)	80	152
Mikolasevic [109]	2022	Croatia	Retrospective	Single-center	135	59 (52–68)	52	135
Younossi [110]	2023	USA	Retrospective	Single-center	463	48	31	463
McPherson [111]	2012	UK	Retrospective	Single-center	123	53 (42–64)	54	123
Sanyal [112]	2023	Global (including US and Europe)	Retrospective	Multicenter	2053	54	62	410
Soresi [113]	2020	Italy	Retrospective	Single-center	57	42 (18–57)	28	57
Kaya [114]	2020	Turkey	Retrospective	Single-center	107	52 (29–71)	36	107
Kawamura [115]	2013	Japan	Retrospective	Single-center	30	60 (29–80)	73	30
Kalaiyarasi [116]	2024	Singapore	Prospective	Single-center	16	49 (18–57)	31	16
Ishiba [117]	2021	Japan	Retrospective	Multicenter	311	58 (16–84)	59	311
Udelsman [118]	2021	USA	Retrospective	Single-center	2465	46 (18–57)	29	2465
Zain [119]	2020	Malaysia	Retrospective	Single-center	122	50 (18–57)	50	122
Lubner [120]	2021	USA	Retrospective	Single-center	186	49	40	186
Soontornmanokul [121]	2013	Thailand	Retrospective	Multicenter	115	51 (18–31)	50	115
Wu [122]	2021	China	Retrospective	Single-center	58	41 (18–57)	85	58
Le [123]	2018	USA	Retrospective	Single-center	254	50	35	254
Sumida [124]	2012	Japan	Retrospective	Multicenter	576	52 (15)	51	576
Chong [125]	2023	Malaysia	Retrospective	Single-center	196	50 (39–61)	50	196
Pérez-Gutiérrez [126]	2013	Mexico and Chile	Retrospective	Multicenter	228	49 (36–61)	49	228
Singh [127]	2017	USA	Retrospective	Multicenter	1157	NR	NR	1157
Panackel [128]	2019	India	Retrospective	Single-center	113	49 (37–61)	55	113
Sanyal [129]	2023	USA	Prospective	Multicenter	1073	54	38	1073
Kolhe [130]	2018	India	Retrospective	Single-center	100	44 (31–56)	42	100
Luger [131]	2016	Austria	Prospective	Single-center	46	42 (13)	20	46

## Appendix C

**Table A3:** Study, patient, and imaging characteristics of included studies evaluating the accuracy of pSWE, 2D-SWE, or VCTE for detecting significant and/or advanced hepatic fibrosis in MASLD patients. pSWE = point shear wave elastography; 2D-SWE = 2D shear wave elastography; VCTE = vibration-controlled elastography; GE = General Electric; NA = not applicable; NR = not reported.

Author	Modality	Year	Country	Study Design	Centers	Vendor Type	Probe Frequency	Min Number of Acquisitions	Technician Experience	Mean Age (Range)	Male Sex (%)	Total Patients
Medellin [132]	pSWE	2019	Canada	Prospective	Single-center	Siemens	NR	10	3–20 years	57 (45–79)	51	47
da Silva [14]	pSWE	2021	Brazil	Prospective	Single-center	Siemens	4–1 MHz	6	>5 years	45	21	79
Roccarina [133]	pSWE	2017	UK	Prospective	Single-center	Philips	NR	NR	NR	56	59	60
Leong [134]	pSWE	2020	Malaysia	Prospective	Single-center	Philips	NR	3	“Experienced”	57 (47–67)	46	100
Kapur [135]	pSWE	2021	India	Prospective	Single-center	Philips	1–5 MHz	NR	NR	39	71	17
Argalia [136]	pSWE	2022	Italy	Prospective	Single-center	Philips	NR	10	>years	52	64	50
Roccarina [137]	pSWE	2022	UK	Retrospective	Single-center	Philips	NR	10	“Experienced”	56 (43–69)	57	159
Taibbi [138]	pSWE	2021	Italy	Prospective	Single-center	Samsung	1–7 MHz	10	>15 years	55 (40–73)	59	46
Cui [139]	pSWE	2016	USA	Prospective	Single-center	Siemens	1–5 MHz	11	0.5 years	49	46	125
Cassinotto [140]	pSWE	2016	France	Prospective	Multicenter	Siemens	NR	10	>2 years	57 (18–80)	59	236
Tomeno [141]	pSWE	2009	Japan	Prospective	Single-center	Siemens	NR	NR	NR	52	NR	50
Yoneda [142]	pSWE	2010	Japan	Prospective	Single-center	Siemens	4 MHz	10	“Experienced”	54	51	NR
Braticevici [143]	pSWE	2013	Romania	Prospective	Single-center	Siemens	4-MHz	10	NR	51 (47–90)	44	64
Lee [144]	2D SWE	2021	Republic of Korea	Prospective	Single-center	Canon	1–8 MHz	9	Radiologist NOS	48 (30–63)	43	102
Zhang [145]	2D SWE	2022	USA	Prospective	Single-center	GE	1–6 MHz	10	>10 years	51.8 (25–78)	46	100
Furlan [146]	2D SWE	2020	USA	Prospective	Single-center	GE	2–5 MHz	10	>10 years	50 (24–53)	42	57
Yu Ogino [147]	2D SWE	2023	Japan	Retrospective	Single-center	GE	NR	6	>26 years	51 (37–65)	61	107
Zhou [148]	2D SWE	2022	China	Retrospective	Single-center	SuperSonic Imagine	NR	5	>10 years	46 (18–77)	47	116
Ozturk [149]	2D SWE	2020	USA	Retrospective	Single-center	SuperSonic Imagine	1–6 MHz	10	Variable	51 (39–62)	47	116
Sharpton [150]	2D SWE	2021	USA	Prospective	Single-center	SuperSonic Imagine	1–6 MHz	3	>1 year	55 (45–64)	54	114
Taru [151]	2D SWE	2024	Romania	Retrospective	Single-center	SuperSonic Imagine	1–6 MHz	5	NR	NR	NR	149
Imajo [152]	2D SWE	2022	Japan	Prospective	Single-center	GE	3–6 MHz	10	>6 years	61 (51–71)	53	201
Herrmann [153]	2D SWE	2018	Multi-country	Retrospective	Multicenter	SuperSonic Imagine	2–6 MHz	1 (variable)	NR	54 (20–83)	54	156
Chen [11]	2D SWE	2022	China	Retrospective	Single-center	SuperSonic Imagine	1–6 MHz	3	NR	32 (16–65)	31	100
Mendoza [154]	2D SWE	2022	Switzerland	Prospective	Single-center	SuperSonic Imagine	NR	3	NR	53 (25–78)	43	88
Takeuchi [13]	2D SWE	2018	Japan	Prospective	Single-center	SuperSonic Imagine	1–6 MHz	5	>10 years	51 (18–82)	65	71
Jamialahmadi [155]	2D SWE	2019	Iran	Prospective	Single-center	SuperSonic Imagine	1–6 MHz	10	NR	39 (27–50)	20	90
Petzold [156]	2D SWE	2020	Germany	Prospective	Single-center	GE	NR	NR	6	53	33	70
Didenko [157]	2D SWE	2019	Ukraine	Prospective	Single-center	Ultrasign	2–5 MHz	NR	Sonographer NOS	47	33	24
Sugimoto [158]	2D SWE	2020	Japan	Prospective	Single-center	Canon	3.5 MHz	10	>15 years	53	51	111
Kalaiyarasi [116]	2D SWE	2024	Singapore	Prospective	Single-center	GE	3–5 MHz	5	>20 years	49	31	16
Seo [159]	2D SWE	2023	Republic of Korea	Prospective	Multicenter	Canon	1–8 MHz	10	NR	36 (27–50)	51	105
Kuroda [160]	2D SWE	2021	Japan	Prospective	Single-center	GE	4.0 MHz	10	Radiologist NOS	55 [18–80]	49	202
Jang [161]	2D SWE	2022	Republic of Korea	Prospective	Multicenter	Canon	1–8 MHz	5	8–28 years	38 (27–54)	48	132
Kim [162]	2D SWE	2022	Republic of Korea	Retrospective	Single-center	Tobshiba	NR	10	NR	51 [25–78]	47	60
Lee [163]	2D SWE	2017	Republic of Korea	Prospective	Single-center	Siemens	1–5 MHz	NR	13 years	56 (53–58)	44	69
Cassinotto [140]	2D SWE	2016	France	Prospective	Multicenter	Siemens	NR	10	>2 years	57 (18–80)	59	236
Duman [15]	VCTE	2024	Turkey	Retrospective	Single center	Echosens	NA	NR	NR	54 (20–73)	32	119
Gabriel-Medina [44]	VCTE	2024	Spain	Retrospective	Single center	Echosens	NA	10	“Experienced”	59	42	140
Mikolasevic [164]	VCTE	2021	Croatia	Prospective	Multicenter	Echosens	NA	10	“Trained”	59	51	179
Eddowes [165]	VCTE	2016	UK	Prospective	Multicenter	Echosens	NA	10	NR	53 (39–66)	57	117
Jun Yang [166]	VCTE	2019	UK	Prospective	Single-center	Echosens	NA	NR	NR	NR	NR	373
Petta [167]	VCTE	2019	UK	Prospective	Multicenter	Echosens	NA	NR	NR	53	57	356
Gitto [168]	VCTE	2021	China	Prospective	Single-center	Echosens	NA	10	“Skilled”	58 (24–74)	40	85
Juan Zhu [169]	VCTE	2020	Japan	Prospective	Multicenter	Echosens	NA	10	“Experienced”	52 (35–70)	58	122
Wong [170]	VCTE	2017	USA	Prospective	Multicenter	Echosens	NA	NR	NR	52	32	292
Kawamura [173]	VCTE	2015	China	Prospective	Single center	Echosens	NA	10	NR	50	54	203
Imajo [152]	VCTE	2017	Japan	Prospective	Single center	Echosens	NA	10	NR	57	50	171
Bae [174]	VCTE	2023	China	Retrospective	Single center	Echosens	NA	10	NR	46 (18–73)	68	71
Park [174]	VCTE	2021	The Netherlands	Prospective	Single center	Echosens	NA	NR	>50 exams	49.5 (20–74)	62	37
Hockings [208]	VCTE	2020	USA	Prospective	Single center	Echosens	NA	10	>100 exams	50 (24–53)	42	59
Chung [175]	VCTE	2022	Republic of Korea	Retrospective	Multicenter	Echosens	NA	10	>500 exams	44 (34–56)	53	251
Costa-Silva [214]	VCTE	2021	USA	Prospective	Single center	Echosens	NA	10	“Trained”	55 (45–64)	54	114
Yilmaz [176]	VCTE	2024	Romania	Retrospective	Single center	Echosens	NA	10	NR	NR	NR	149
Bahl [177]	VCTE	2022	Japan	Retrospective	Multicenter	Echosens	NA	NR	NR	NR	NR	126
Pan [178]	VCTE	2022	Japan	Prospective	Single center	Echosens	NA	10	6 years	61 (51–71)	53	201
Lu [179]	VCTE	2017	UK	Prospective	Single center	Echosens	NA	NR	NR	56	59	60
Fujii [180]	VCTE	2022	Switzerland	Prospective	Single-center	Echosens	NA	3	NR	53 [25–78]	58	102
Machado [181]	VCTE	2024	Singapore	Prospective	Single-center	Echosens	NA	10	NR	49	5	16
Tokushige [182]	VCTE	2023	Republic of Korea	Retrospective	Multicenter	Echosens	NA	NR	NR	36 (27–50)	51	105
Yang [184]	VCTE	2021	Japan	Prospective	Single-center	Echosens	NA	NR	“Experienced”	55 (18–80)	49	202
Ruiz-Fernandez [185]	VCTE	2022	Republic of Korea	Retrospective	Single-center	Echosens	NA	NR	10	51 (25–78)	47	60
Del Barrio Azaceta [186]	VCTE	2016	Republic of Korea	Prospective	Single-center	Echosens	NA	NR	>1000 exam	56 (53–58)	44	94
Hernandez-Rocha [187]	VCTE	2020	Malaysia	Prospective	Single-center	Echosens	NA	10	“Trained”	57 (47–67)	46	100
Zheng [188]	VCTE	2021	Italy	Prospective	Single-center	Echosens	NA	10	>15 years	55 (40–73)	59	46
Chuah [189]	VCTE	2022	Italy	Prospective	Single-center	Echosens	NA	10	NR	52	64	50
Ghanvatkar [190]	VCTE	2022	UK	Retrospective	Single center	Echosens	NA	10	“Experienced”	56	NR	159
Chu [191]	VCTE	2017	USA	Prospective	Single-center	Echosens	NA	10	NR	51	43	94
Yang [192]	VCTE	2016	Japan	Prospective	Multicenter	Echosens	NA	10	NR	58	57	127
Roh [193]	VCTE	2018	Japan	Retrospective	Single-center	Echosens	NA	NR	NR	54	58	113
Bob Harrap [194]	VCTE	2023	Italy	Prospective	Single center	Echosens	NA	10	“Experienced”	50 (20–74)	62	96
de Ledinghen [195]	VCTE	2010	HK, France	Prospective	Multicenter	Echosens	NA	10	NR	51	55	246
Garteiser [196]	VCTE	2016	France	Retrospective	Multicenter	Echosens	NA	10	“Experienced”	56	57	452
Gaia [197]	VCTE	2023	Iran	Prospective	Multicenter	Echosens	NA	10	>500 exams	43	62	73
Alsaqal [198]	VCTE	2010	Malaysia	Prospective	Single-center	Echosens	NA	NR	NR	49	60	25
Naveau [199]	VCTE	2023	Italy	Retrospective	Single center	Echosens	NA	10	“Experienced”	52 (25–78)	65	520
Jafarov [200]	VCTE	2017	Hong Kong	Prospective	Single-center	Echosens	NA	10	>50 exams	52 (41–57)	55	215
Tapper [201]	VCTE	2023	Multi-country	Prospective	Multicenter	Echosens	NA	NR	NR	51	58	632
Chakraborty [202]	VCTE	2021	Brazil	Prospective	Single-center	Echosens	NA	10	NR	36 (20–67)	31	85
Siddiqui [203]	VCTE	2016	Republic of Korea	Prospective	Multicenter	Echosens	NA	10	“Experienced”	41	61	183
Pathik [204]	VCTE	2019	UK	Retrospective	Single-center	Echosens	NA	10	NR	47 (37–57)	81	27
Ergelen [205]	VCTE	2018	Australia	Prospective	Multi-center	Echosens	NA	10	>2000 exams	46	32	66
Kosick [206]	VCTE	2019	Austria	Prospective	Multicenter	Echosens	NA	10	NR	52	57	140
Tovo [207]	VCTE	2023	China	Retrospective	Single-center	Echosens	NA	NR	NR	43 (35–59)	59	172
Li [67]	VCTE	2012	Canada	Prospective	Multicenter	Echosens	NA	10	“Experienced”	50 (43–57)	63	75
Bhatia [104]	VCTE	2023	Greece	Retrospective	Single-center	Echosens	NA	10	>10 years	50 (16–69)	60	38
Koh [105]	VCTE	2020	Taiwan	Prospective	Single-center	Echosens	NA	10	"Trained"	36	29	123
Boursier [48]	VCTE	2023	France	Prospective	Multicenter	Echosens	NA	NR	NR	59 (median)	58	163
Bertot [49]	VCTE	2022	Spain	Prospective	Single-center	Echosens	NA	NR	NR	51	40	115
Al-Fryan [24]	VCTE	2019	Italy, France, HK, China	Prospective	Multicenter	Echosens	NA	10	>300 exams	50	63	968
Chawla [25]	VCTE	2023	Spain	Prospective	Multicenter	Echosens	NA	10	NR	56	66	1124
Hernandez [26]	VCTE	2015	China	Prospective	Multicenter	Echosens	NA	10	>300 exams	57 (47–67)	46	101
Pópulo [27]	VCTE	2023	France	Prospective	Multicenter	Echosens	NA	10	NR	58 (50–66)	60	1051
Jeong [28]	VCTE	2021	USA	Retrospective	Multicenter	Echosens	NA	NR	NR	57 (median)	NR	1308
Kwee [29]	VCTE	2023	USA	Retrospective	Multicenter	Echosens	NA	10	“Trained”	58	35	548
Trifan [30]	VCTE	2014	USA	Prospective	Single-center	Echosens	NA	NR	NR	54 (44–64)	40	94
Gaia-Póvoa [31]	VCTE	2016	France	Prospective	Multicenter	Echosens	NA	10	“Experienced”	57 (18–80)	59	223
Boursier [32]	VCTE	2020	Malaysia	Retrospective	Single-center	Echosens	NA	10	“Trained”	55	45	136
Younes [33]	VCTE	2023	Malaysia/HK/China	Prospective	Multicenter	Echosens	NA	10	NR	48	57	396
Marra [34]	VCTE	2015	Turkey	Prospective	Single-center	Echosens	NA	10	NR	46 (24–62)	49	87
Liu [35]	VCTE	2017	India	Retrospective	Single-center	Echosens	NA	10	NR	44	75	779
Yoo [36]	VCTE	2010	Japan	Prospective	Single-center	Echosens	NA	10	“Experienced”	51	46	54
Saadeh [37]	VCTE	2008	Japan	Prospective	Multicenter	Echosens	NA	10	NR	52 [25–78]	41	97
Watanabe [38]	VCTE	2019	Multicenter	Prospective	Multicenter	Echosens	NA	10	NR	59 (53–65)	58	1765
Xiao [39]	VCTE	2021	China	Prospective	Single-center	Echosens	NA	10	“Experienced”	40 (32–56)	51	91
Cai [40]	VCTE	2013	Malaysia	Prospective	Single-center	Echosens	NA	10	NR	50 (38–62)	53	120
Barritt [41]	VCTE	2010	Romania	Prospective	Single-center	Echosens	NA	10	NR	42 (20–69)	71	72
Harrison [42]	VCTE	2023	India, Singapore	Retrospective	Multicenter	Echosens	NA	10	“Trained”	43	55	641
Petta [43]	VCTE	2009	France/China	Prospective	Multicenter	Echosens	NA	NR	NR	51	55	208
Selvarajah [45]	VCTE	2018	Germany	Prospective	Single-center	Echosens	NA	10	“Trained”	51 (19–93)	53	126
Djordjevic [46]	VCTE	2017	Republic of Korea	Prospective	Multicenter	Echosens	NA	10	>1000 exams	56 (53–58)	44	94
Sanyal [47]	VCTE	2021	France	Prospective	Single-center	Echosens	NA	10	>100 exams	42 ± 11	16	152
Aziz [50]	VCTE	2011	Italy	Prospective	Single-center	Echosens	NA	10	“Trained”	48 (24–65)	72	72
Ruiz [51]	VCTE	2019	USA	Retrospective	Multicenter	Echosens	NA	10	“Experienced”	59	45	1549
Jang [52]	VCTE	2020	USA	Prospective	Multi-center	Echosens	NA	10	NR	55	62	212
Sung [53]	VCTE	2015	Italy	Prospective	Multicenter	Echosens	NA	10	NR	45 (18–78)	70	321
Kim [54]	VCTE	2022	Sweden	Prospective	Single-center	Echosens	NA	10	“Experienced”	55 (13.09)	59	66
Liang [55]	VCTE	2014	France	Prospective	Single-center	Echosens	NA	10	NR	43	19	100
Zhang [56]	VCTE	2020	Turkey	Prospective	Single-center	Echosens	NA	10	>15,000 exams	49	59	139
Wang [57]	VCTE	2016	USA	Prospective	Single-center	Echosens	NA	10	>500 exams	51	59	120
Cho [58]	VCTE	2019	USA	Retrospective	Single-center	Echosens	NA	10	NR	54		191
Toubia [59]	VCTE	2019	USA	Prospective	Multicenter	Echosens	NA	10	“Trained”	51	32	393
Kim [60]	VCTE	2015	India	Prospective	Single-center	Echosens	NA	10	NR	42 (18–80)	46	110
Nam [61]	VCTE	2023	Multiple	Not specified	Multicenter	Echosens	NA	8	NR	55	51	1434
Moon [62]	VCTE	2019	France	Prospective	Multicenter	Echosens	NA	10	NR	57 (18–80)	59	938
Kim [63]	VCTE	2023	Australia	Retrospective	Single-center	Echosens	NA	10	“Experienced”	52 (40–64)	40	125
Park [64]	VCTE	2016	Turkey	Prospective	Single-center	Echosens	NA	10	NR	47 (25–78)	62	63
Cho [65]	VCTE	2019	Canada	Retrospective	Single-center	Echosens	NA	NR	NR	55 (50–64)	54	86
Jeong [66]	VCTE	2024	Hong Kong	Retrospective	Single	Echosens	NA	10	“Experienced”	52 (18–57)	55	431
Li [67]	VCTE	2019	Brazil	Prospective	Multicenter	Echosens	NA	10	>500 exams	55 (45–65)	26	104

## Appendix D

**Table A4:** Study, patient, and imaging characteristics of included studies evaluating the accuracy of MRE for detecting significant and/or advanced hepatic fibrosis in MASLD patients. MRE = magnetic resonance elastography; NR = not reported; GE = General Electric; GRE = gradient echo; SE-EPI = spin echo echoplanar; ROI = region of interest.

Author	Year	Country	Study Design	Centers	Vendor Type	Reader Experience	MRI Field Strength	Pulse Sequence	Driver Amplitude	Segmentation Used	Mean Age (Range)	Male Sex (%)	Total Patients
Duman [15]	2024	Turkey	Retrospective	Single-center	Siemens	NR	1.5T	2D GRE	NR	Manual ROI	54 (20–73)	32	119
Troelstra [172]	2021	The Netherlands	Prospective	Single-center	Phillips	3 years	3T	2D GRE	50 Hz	Manual ROI	49	62	35
Hockings [208]	2020	Sweden	NR	Not specified	NR	NR	NR	NR	NR	NR	NR	NR	68
Cui [16]	2015	USA	Prospective	Single-center	GE	NR	3T	2D GRE	NR	Manual ROI	51	41	102
Zhang [145]	2022	USA	Prospective	Single-center	GE	>1 year	3T	2D GRE	60 Hz	Manual ROI	52 (25–78)	46	100
Furlan [146]	2020	USA	Prospective	Single-center	NR	NR	1.5T	2D GRE	NR	NR	50 (24–53)	42	59
Imajo [152]	2022	Japan	Prospective	Single-center	GE	>6 years	3T	SE-EPI	60 Hz	Manual ROI	61 (51–71)	53	201
Kalaiyarasi [116]	2024	Singapore	Prospective	Single-center	GE	“Experienced”	1.5T	2D GRE	NR	Manual ROI	49	31	16
Loomba [209]	2013	USA	Prospective	Single-center	NR	NR	NR	NR	NR	NR	50	52	52
Loomba [210]	2014	USA	Prospective	Single-center	GE	“Trained”	3T	2D GRE	60 Hz	Manual ROI	50	44	117
Loomba [211]	2016	USA	Prospective	Single-center	GE	“Trained”	3T	2D GRE	60 Hz	Manual ROI	50	44	99
Loomba [212]	2020	USA	Retrospective	Multicenter	NR	NR	NR	NR	NR	NR	NR	44	296
Li [213]	2023	USA	Prospective	Single-center	GE	5 years	1.5T	2D GRE	60 Hz	Manual ROI	55 (46–60)	38	104
Cui [139]	2016	USA	Prospective	Single-center	GE	NR	3T	2D GRE	NR	Manual ROI	49 (15.4)	46	125
Park [174]	2017	USA	Prospective	Single-center	GE	“Trained”	3T	2D GRE	60 Hz	Manual ROI	51	43	94
Imajo [175]	2016	Japan	Prospective	Multicenter	GE	Radiologist	3T	SE-EPI	NR	Manual ROI	58	57	142
Costa-Silva [214]	2018	Brazil	Prospective	Single-center	GE	15 years	1.5T	NR	60 Hz	Manual ROI	54 (25–76)	14	49
Kim [215]	2020	Republic of Korea	Prospective	Single-center	Siemens	6–25 years	3T	SE-EPI	NR	NR	51	34	47
Hanniman [216]	2022	Canada	Prospective	Single-center	GE	Medical student, Fellow	3T	NR	60 Hz	Manual ROI	55 (33–74)	37	49
Alsaqal [198]	2022	Sweden	Prospective	Single-center	GE	“Trained”	3T	2D GRE	NR	NR	55 (18–70)	59	64
Naveau [199]	2023	USA/Japan	Prospective	Multicenter	GE	“Trained”	1.5T and 3T	NR	NR	NR	57 (46–67)	48	806
Jafarov [200]	2022	Japan	Retrospective	Single-center	GE	NR	1.5T	2D GRE	60 Hz	Manual ROI	65 (58–72)	45	105
Tapper [201]	2018	Japan	Retrospective	Single-center	NR	NR	NR	NR	NR	NR	54	58	111
Chakraborty [202]	2021	USA/Japan	Prospective	Multi-center	GE	Radiologist	3T	NR	60Hz	Automated ROI	51 (USA)/56 (Japan)	46 (USA)/56 (Japan)	238 (USA)/272 (Japan)

## Appendix E

**Table A5:** Results of QUADAS-2 assessment for risk of bias and applicability concerns reported on a per index test (modality) basis.

Author	Modality	RoB Patient Selection	RoB Index Test	RoB Reference Standard	RoB Flow and Timing	AC Patient Selection	AC Index Test	AC Reference Standard
Lee	2D SWE	Low	High	Low	Low	Low	Low	Low
Zhang	2D SWE	Low	High	Low	Low	Low	Low	Low
Furlan	2D SWE	Low	High	Low	Low	Low	Low	Low
Yu Ogino	2D SWE	Low	High	Low	High	High	Low	Low
Zhou	2D SWE	Low	High	Low	Low	Low	High	Low
Ozturk	2D SWE	High	High	Low	Low	Low	Low	Low
Sharpton	2D SWE	High	High	Low	Low	Low	Low	Low
Taru	2D SWE	Low	High	Unclear	Unclear	Low	Unclear	Low
Imajo	2D SWE	Low	High	Low	Low	Low	Low	Low
Herrmann	2D SWE	Low	High	Low	Low	Low	Low	Low
Chen	2D SWE	Low	High	Low	Low	Low	Low	Low
Mendoza	2D SWE	Low	Low	Low	Low	Low	Low	Low
Takeuchi	2D SWE	Low	High	Low	Low	Low	Low	Low
Jamialahmadi	2D SWE	High	High	High	High	High	High	Low
Petzold	2D SWE	Low	High	Low	High	Low	Low	Low
Didenko	2D SWE	Low	Unclear	Low	Low	Low	Low	Low
Sugimoto	2D SWE	High	High	Low	High	High	High	Low
Kalaiyarasi	2D SWE	High	Low	Low	High	High	Low	Low
Seo	2D SWE	High	High	High	Low	High	High	High
Kuroda	2D SWE	Low	High	Low	Low	Low	Low	Low
Jang	2D SWE	Low	Unclear	High	Low	Low	Low	High
Kim	2D SWE	Low	High	Low	Low	Low	Unclear	Low
Lee	2D SWE	Low	High	Low	Low	Low	Low	Low
Cassinotto	2D SWE	Low	High	Low	Low	Low	High	Low
Medellin	pSWE	Low	Low	Low	Unlear	Low	Low	Low
da Silva	pSWE	Low	High	Low	Low	Low	High	Low
Roccarina	pSWE	Low	High	Unclear	Unclear	Unclear	High	Unclear
Leong	pSWE	Low	High	Low	Low	Low	High	Low
Kapur	pSWE	High	High	High	Yes	Low	High	High
Argalia	pSWE	Low	High	Low	Low	Low	High	Low
Roccarina	pSWE	Low	High	High	High	Low	High	Low
Taibbi	pSWE	Low	High	Low	Low	Low	High	Low
Cui	pSWE	Low	High	Low	Low	Low	High	Low
Cassinotto	pSWE	Low	High	Low	Low	Low	High	Low
Tomeno	pSWE	Low	High	Low	Low	Low	High	Low
Yoneda	pSWE	Low	High	Low	High	Low	High	Low
Braticevici	pSWE	Low	High	Low	Low	Low	Low	Low
Duman	VCTE	Low	High	Low	Low	Low	High	Low
Gabriel-Medina	VCTE	Low	High	Low	Low	Low	High	Low
Mikolasevic	VCTE	Low	High	Low	Low	Low	High	Low
Eddowes	VCTE	Low	High	Low	Low	Low	High	Low
Eddowes	VCTE	Low	High	Low	Low	Low	High	Low
Eddowes	VCTE	Low	High	Low	Low	Low	High	Low
Yu	VCTE	Low	High	Low	Low	Low	High	Low
Oeda	VCTE	Low	High	Low	Low	Low	High	Low
Siddiqui	VCTE	Low	High	Low	Low	Low	High	Low
Wong	VCTE	Low	High	Unclear	Low	Unclear	High	Unclear
Seki	VCTE	Low	High	Low	Low	Low	High	Low
Zhang	VCTE	Low	High	Low	Low	Low	High	Low
Troelstra	VCTE	Low	High	Low	Low	Low	High	Low
Furlan	VCTE	Low	High	Low	Low	Low	High	Low
Lee	VCTE	Low	High	Unclear	Low	Low	High	Low
Sharpton	VCTE	High	High	Low	Low	Low	High	Low
Taru	VCTE	Low	High	Low	Low	Low	High	Low
Kawamura	VCTE	Low	Low	Low	Low	Low	Low	Low
Imajo	VCTE	Low	High	Low	Low	Low	High	Low
Roccarina	VCTE	High	High	Low	Low	High	High	Low
Mendoza	VCTE	Low	High	Low	Low	Low	High	Low
Kalaiyarasi	VCTE	High	High	Low	High	High	High	Low
Seo	VCTE	High	High	High	Low	High	High	High
Kuroda	VCTE	Low	High	Low	Low	Low	High	Low
Kim	VCTE	Low	High	Low	Low	Low	High	Low
Lee	VCTE	Low	High	Low	Low	Low	High	Low
Leong	VCTE	High	High	High	Low	High	High	Low
Taibbi	VCTE	Low	High	Low	Low	Low	High	Low
Argalia	VCTE	Yes	High	Yes	Yes	Low	High	Low
Roccarina	VCTE	Low	High	Low	Unclear	Low	High	Low
Park	VCTE	Low	High	High	Low	Low	High	Low
Imajo	VCTE	Yes	High	High	Unclear	Low	High	Low
Ogawa	VCTE	High	High	Low	Low	High	High	Low
Armandi	VCTE	Low	High	Low	Low	Low	High	Low
Wong	VCTE	Low	High	Low	Low	Low	High	Low
Boursier	VCTE	Low	High	Low	Low	Low	High	Low
Salehi	VCTE	Low	High	Low	Low	Low	High	Low
Mahadeva	VCTE	Low	High	Low	Low	Low	High	Low
Pennisi	VCTE	Low	Low	Low	Low	Low	Low	Low
Loong	VCTE	Low	Unclear	Low	Low	Low	Unclear	Low
Vali	VCTE	Low	High	Low	Low	Low	High	Low
Filho	VCTE	Low	High	Low	High	Low	High	Low
Lee	VCTE	Low	High	Low	Low	Low	High	Low
Prat	VCTE	Low	Unclear	Low	Low	Low	Unclear	Low
Ooi	VCTE	Low	High	Low	Low	Low	High	Low
Staufer	VCTE	Low	High	Low	Low	Low	High	Low
Lu	VCTE	High	High	Low	Low	High	High	Low
Myers	VCTE	Low	High	Low	Low	Low	High	Low
Arvaniti	VCTE	Unclear	High	Low	Low	Unclear	High	Unclear
Kao	VCTE	Low	High	Low	Low	Low	High	Low
Castera	VCTE	Low	Low	Low	Low	Low	Low	Low
Ruiz-Fernandez	VCTE	Low	High	Low	Low	Low	High	Low
Petta	VCTE	Low	Low	Low	Low	Low	Low	Low
Del Barrio Azaceta	VCTE	Low	Unclear	Low	Unclear	Low	Unclear	Low
Shen	VCTE	Low	High	Low	Low	Low	High	Low
Boursier	VCTE	Low	Low	Low	Low	Low	Low	Low
Noureddin	VCTE	Unclear	Unclear	Unclear	Unclear	Unclear	Unclear	Unclear
Noureddin	VCTE	Low	Low	Low	Low	Low	Low	Low
Vuppalanchi	VCTE	Unclear	High	Low	High	Unclear	High	Low
Cassinotto	VCTE	Low	High	Low	Low	Low	High	Low
Chuah	VCTE	Low	Unclear	Low	Low	Low	Unclear	Low
Cheung	VCTE	Low	High	Low	Low	Low	High	Low
Ergelen	VCTE	Low	High	Low	Low	Low	High	Low
Bhadoria	VCTE	Low	High	Low	Low	Low	High	Low
Yoneda	VCTE	Low	High	Low	Low	Low	High	Low
Yoneda	VCTE	Low	High	Low	Low	Low	High	Low
Anstee	VCTE	Low	Low	Low	Low	Low	Low	Low
Yang	VCTE	Low	High	Low	Low	Low	High	Low
Mahadeva	VCTE	Low	High	Low	Low	Low	High	Low
Lupsor	VCTE	Low	High	Low	Low	Low	High	Low
Arora	VCTE	Low	High	Low	Low	Low	High	Low
de Ledinghen	VCTE	Low	High	Low	Low	Low	High	Low
Labenz	VCTE	Low	Unclear	Low	High	Low	Unclear	Low
Lee	VCTE	Low	High	Low	Low	Low	High	Low
Garteiser	VCTE	Low	High	Low	Low	Low	High	Low
Gaia	VCTE	High	High	Low	Low	High	High	Low
Barritt	VCTE	Unclear	Unclear	Low	Unclear	Unclear	Unclear	Low
Harrison	VCTE	Low	High	Low	Low	Low	High	Low
Petta	VCTE	Low	Unclear	Low	Low	Low	Unclear	Low
Alsaqal	VCTE	Low	High	Low	Low	Low	High	Low
Naveau	VCTE	Low	High	Low	Low	Low	High	Low
Jafarov	VCTE	Low	High	Low	Low	Low	High	Low
Tapper	VCTE	Low	High	Low	Low	Low	High	Low
Chakraborty	VCTE	Low	High	Low	Low	Low	High	Low
Siddiqui	VCTE	Low	High	Low	Low	Low	High	Low
Pathik	VCTE	Low	High	Low	Low	Low	High	Low
Sanyal	VCTE	Low	High	Low	Low	Low	High	Low
Boursier	VCTE	Low	High	Low	Low	Low	High	Low
Bertot	VCTE	Low	Low	Low	Low	Low	Low	Low
Ergelen	VCTE	Unclear	High	Low	Low	Unclear	High	Low
Kosick	VCTE	Unclear	High	Unclear	Unclear	Unclear	High	Unclear
Li	VCTE	Low	Low	Unclear	Low	Low	Low	Unclear
Tovo	VCTE	Low	Low	Low	Low	Low	Low	Low
Duman	MRE	Low	High	Low	Low	Low	High	Low
Troelstra	MRE	Low	High	Low	Low	Low	High	Low
Hockings	MRE	Unclear	Unclear	Unclear	Unclear	Unclear	Unclear	Unclear
Cui	MRE	Low	Low	Low	Low	Low	Low	Low
Zhang	MRE	Low	High	Low	Low	Low	High	Low
Furlan	MRE	Low	High	Low	Unclear	Low	High	Low
Imajo	MRE	Low	High	Low	Low	Low	High	Low
Kalaiyarasi	MRE	Low	Low	Low	Low	Low	Low	Low
Loomba	MRE	Unclear	Unclear	Low	Unclear	Unclear	Unclear	Low
Loomba	MRE	Low	High	Low	Low	Low	High	Low
Loomba	MRE	Low	Unclear	Low	Low	Low	Unclear	Low
Loomba	MRE	Low	Low	Low	Unclear	Low	Low	Low
Li	MRE	Low	High	Low	Low	Low	High	Low
Cui	MRE	Low	High	Low	Low	Low	High	Low
Park	MRE	Low	High	Low	Low	Low	High	Low
Imajo	MRE	Low	High	Low	Low	Low	High	Low
Costa-Silva	MRE	Low	High	Low	Unclear	Low	High	Low
Kim	MRE	Low	High	Low	Low	Low	High	Low
Hanniman	MRE	Low	Low	Low	Low	Low	Low	Low
Alsaqal	MRE	Low	High	Low	Low	Low	High	Low
Tamaki	MRE	Low	Low	Low	Low	Low	Low	Low
Inada	MRE	Low	High	Low	Low	Low	High	Low
Ogawa	MRE	High	High	Low	Low	High	High	Low
Jung	MRE	Low	High	Low	Low	Low	High	Low
Chen	FIB-4	Unclear	Unclear	Unclear	Unclear	Unclear	Unclear	Unclear
Lee	FIB-4	Low	Low	Low	Low	Low	Low	Low
Takeuchi	FIB-4	Low	Low	Low	Low	Low	Low	Low
da Silva	FIB-4	Low	Low	Low	Low	Low	Low	Low
Duman	FIB-4	Unclear	Low	Low	Low	Low	Low	Low
Cui	FIB-4	Low	Low	Low	Low	Low	Low	Low
Inada	FIB-4	Low	High	Low	Low	High	High	Low
Tamaki	FIB-4	Low	Low	Low	Low	High	High	Low
Ogawa	FIB-4	Unclear	High	Low	Unclear	Unclear	High	Low
Jung	FIB-4	Low	Low	Low	Low	Low	Low	Low
Armandi	FIB-4	Low	Low	Low	Unclear	Low	Low	Low
Wong	FIB-4	Low	Low	Low	Low	Low	Low	Low
Boursier	FIB-4	Low	Low	Low	Low	Low	Low	Low
Pennisi	FIB-4	Low	Low	Low	Low	Low	Low	Low
Pennisi	FIB-4	Low	Low	Low	Low	Low	Low	Low
Prat	FIB-4	Low	Low	Low	Low	Low	Low	Low
Arvaniti	FIB-4	Low	Low	Low	Low	Low	Low	Low
Kao	FIB-4	Unclear	High	Unclear	Low	Unclear	Unclear	Unclear
Castera	FIB-4	Low	Unclear	Low	Low	Low	Unclear	Low
Petta	FIB-4	Low	Low	Low	Unclear	Low	Low	Low
Staufer	FIB-4	Low	Low	Low	Low	Low	Low	Low
Boursier	FIB-4	Low	Low	Low	Low	Low	Low	Low
Noureddin	FIB-4	Low	Low	Low	Low	Low	Low	Low
Noureddin	FIB-4	Low	Unclear	Low	Low	Low	Unclear	Low
Cheung	FIB-4	Low	Unclear	Low	Low	Low	Unclear	Low
Bhadoria	FIB-4	Low	Unclear	Unclear	Low	Low	Unclear	Unclear
Anstee	FIB-4	Low	Unclear	Low	Unclear	Low	Unclear	Low
Anstee	FIB-4	Low	High	Low	Low	Low	High	Low
Labenz	FIB-4	Low	Low	Low	Low	Low	Low	Low
Arora	FIB-4	Low	Unclear	Unclear	Low	Low	Unclear	Unclear
Barritt	FIB-4	Unclear	Unclear	Low	Unclear	Unclear	Unclear	Low
Harrison	FIB-4	Low	Low	Low	Low	Low	Low	Low
Petta	FIB-4	Low	Low	Low	Low	Low	Low	Low
Gabriel-Medina	FIB-4	Low	Low	Low	Low	Low	Low	Low
Zhang	FIB-4	Low	Low	Low	Low	Low	Low	Low
Eddowes	FIB-4	Low	Low	Low	Low	Low	Low	Low
Sanyal	FIB-4	Low	Low	Low	Low	Low	Low	Low
Sanyal	FIB-4	Low	Low	Low	Low	Low	Low	Low
Sanyal	FIB-4	Low	Low	Low	Low	Low	Low	Low
Boursier	FIB-4	Low	Low	Low	Low	Low	Low	Low
Bertot	FIB-4	Low	Low	Low	Low	Low	Low	Low
Kobayashi	FIB-4	Low	Unclear	Low	Low	Low	Unclear	Low
Eren	FIB-4	Low	Low	Low	Low	Low	Low	Low
Singh	FIB-4	Low	Low	Low	Low	Low	Low	Low
Marella	FIB-4	High	Low	Low	Unclear	High	Low	Low
Treeprasertsuk	FIB-4	Low	Low	Low	Unclear	Low	Low	Low
McPherson	FIB-4	Low	Low	Low	Low	Low	Low	Low
McPherson	FIB-4	Low	Low	Low	Low	Low	Low	Low
Kolhe	FIB-4	High	Low	Low	Low	High	Low	Low
Kaya	FIB-4	Low	Low	Low	Low	Low	Low	Low
Nones	FIB-4	Low	Unclear	Low	Low	Low	Unclear	Low
Balakrishnan	FIB-4	Unclear	Unclear	Unclear	Unclear	Unclear	Unclear	Unclear
Alkayyali	FIB-4	Low	Low	Low	Low	Low	Low	Low
Alkayyali	FIB-4	Low	Low	Low	Low	Low	Low	Low
Nielsen	FIB-4	Low	Low	Low	Low	Low	Low	Low
Ampuero	FIB-4	Low	Low	Low	Low	Low	Low	Low
Sang	FIB-4	Low	Low	Low	Low	Low	Low	Low
Shima	FIB-4	Low	Low	Low	Low	Low	Low	Low
Seko	FIB-4	Low	Low	Low	Low	Low	Low	Low
Siddiqui	FIB-4	Low	Low	Low	Low	Low	Low	Low
Li	FIB-4	Low	Low	Low	Low	Low	Low	Low
Li	FIB-4	Low	Low	Low	Low	Low	Low	Low
Sanyal	FIB-4	Low	Low	Low	Low	Low	Low	Low
Moon	FIB-4	Low	Low	Low	Low	Low	Low	Low
Zambrano-Huailla	FIB-4	Unclear	Unclear	Low	Low	Unclear	Unclear	Low
Yang	FIB-4	High	Unclear	Low	Unclear	High	Unclear	Low
Giamarrino	FIB-4	Unclear	Unclear	Unclear	Unclear	Unclear	Unclear	Unclear
Nishikawa	FIB-4	Low	Low	Low	Low	Low	Low	Low
Kakisaka	FIB-4	Low	Low	Low	Unclear	Low	Low	Low
Schmitz	FIB-4	Low	Low	Low	Low	Low	Low	Low
Zhang	FIB-4	Low	Low	Low	Low	Low	Low	Low
Balakrishnan	FIB-4	Low	Low	Low	Low	Low	Low	Low
Kariyama	FIB-4	Low	Unclear	Low	Low	Low	Unclear	Low
Mohammed	FIB-4	Low	Low	Low	Low	Low	Low	Low
de la Tijera	FIB-4	Low	Low	Low	Low	Low	Low	Low
McPherson	FIB-4	Low	Low	Low	Low	Low	Low	Low
Shah	FIB-4	Low	Low	Low	Low	Low	Low	Low
Maurice	FIB-4	Low	Unclear	Low	Unclear	Low	Low	Low
Zhou	FIB-4	Low	Low	Low	Unclear	Low	Low	Low
Kouvari	FIB-4	Low	Low	Low	Low	Low	Low	Low
Ballestri	FIB-4	Low	Low	Low	Low	Low	Low	Low
Noureddin	FIB-4	Low	Low	Low	Low	Low	Low	Low
Singh	FIB-4	Unclear	Unclear	Unclear	Unclear	Unclear	Unclear	Unclear
Prasad	FIB-4	Unclear	Unclear	Yes	Unclear	Unclear	Unclear	Low
Kim	FIB-4	Low	Low	Low	Unclear	Low	Low	Low
Yoneda	FIB-4	Low	Low	Low	Unclear	Low	Low	Low
Qadri	FIB-4	Low	Unclear	Low	Low	Low	Unclear	Low
Miller	FIB-4	Low	Unclear	Unclear	Unclear	Low	Unclear	Unclear
Drolz	FIB-4	Low	Low	Unclear	Unclear	Low	Low	Unclear
Meneses	FIB-4	Low	Low	Low	Low	Low	Low	Low
Alqahtani	FIB-4	Unclear	Low	Low	Unclear	Unclear	Low	Low
De Carli	FIB-4	Unclear	High	Unclear	High	Unclear	High	Unclear
Ito	FIB-4	Unclear	Unclear	Unclear	Unclear	Unclear	Unclear	Unclear
Bril	FIB-4	Low	Low	Low	Unclear	Low	Low	Low
Satapathy	FIB-4	Unclear	Unclear	Unclear	Unclear	Unclear	Unclear	Unclear
Moon	FIB-4	Unclear	Low	Low	Unclear	Unclear	Low	Low
Schwenger	FIB-4	High	Low	Low	Unclear	High	Low	Low
Andrade	FIB-4	Low	Unclear	Low	Unclear	Low	Unclear	Low
Aida	FIB-4	Low	Unclear	Unclear	Low	Low	Low	Low
Huang	FIB-4	High	Unclear	Low	Unclear	High	Unclear	Low
McPherson	FIB-4	Unclear	Unclear	Unclear	Low	Unclear	Unclear	Unclear
Kaya	FIB-4	Low	Low	Low	Unclear	Low	Low	Low
Xun	FIB-4	Low	Low	Low	Low	Low	Low	Low
Mikolasevic	FIB-4	Unclear	Unclear	Unclear	Unclear	Unclear	Unclear	Unclear
Younossi	FIB-4	Low	Low	Unclear	Low	Low	Low	Low
McPherson	FIB-4	Unclear	Unclear	Unclear	Unclear	Unclear	Unclear	Unclear
Sanyal	FIB-4	Low	Low	Low	Low	Low	Low	Low
Soresi	FIB-4	Low	High	Low	Low	Low	High	Low
Kaya	FIB-4	Low	Low	Low	Low	Low	Low	Low
Kawamura	FIB-4	Low	Unclear	Unclear	Low	Low	Low	Low
Kalaiyarasi	FIB-4	Unclear	Low	Low	Unclear	Unclear	Low	Low
Ishiba	FIB-4	Unclear	Low	Unclear	Unclear	Unclear	Low	Unclear
Udelsman	FIB-4	Low	Unclear	Unclear	Unclear	Low	Low	Unclear
Zain	FIB-4	Unclear	Unclear	Unclear	Unclear	Unclear	Unclear	Unclear
Lubner	FIB-4	Unclear	Low	Low	Low	Unclear	Low	Low
Soontornmanokul	FIB-4	Unclear	Low	Unclear	Unclear	Unclear	Low	Unclear
Wu	FIB-4	Low	Low	Low	Low	Low	Low	Low
Le	FIB-4	Unclear	Unclear	Low	Unclear	Unclear	Unclear	Low
Sumida	FIB-4	Unclear	Low	Low	Unclear	Unclear	Low	Low
Chong	FIB-4	High	Unclear	Unclear	Unclear	High	Unclear	Unclear
Pérez-Gutiérrez	FIB-4	Unclear	Low	Unclear	Unclear	Unclear	Low	Unclear
Singh	FIB-4	Unclear	Low	Unclear	Unclear	Unclear	Low	Unclear
Panackel	FIB-4	High	Unclear	Unclear	Unclear	High	Unclear	Low
Sanyal	FIB-4	High	Unclear	Low	Unclear	High	Unclear	Low
Kolhe	FIB-4	Low	Unclear	Unclear	Low	Low	Unclear	Unclear
Luger	FIB-4	Unclear	Unlcear	Unclear	Unclear	Low	Low	Low
